# Machine learning combined with CT-based radiomics predicts the prognosis of oesophageal squamous cell carcinoma

**DOI:** 10.1186/s13244-025-02049-8

**Published:** 2025-10-01

**Authors:** Mingyu Liu, Rongxin Lu, Bo Wang, Jun Fan, Yuheng Wang, Jiashan Zhu, Jinhua Luo

**Affiliations:** Department of Thoracic Surgery, The First Affiliated Hospital of Nanjing Medical University, Nanjing, China

**Keywords:** Oesophageal squamous cell carcinoma, Machine learning, Radiomics, Personalised medicine

## Abstract

**Objectives:**

This retrospective study aims to develop a machine learning model integrating preoperative CT radiomics and clinicopathological data to predict 3-year recurrence and recurrence patterns in postoperative oesophageal squamous cell carcinoma.

**Materials and methods:**

Tumour regions were segmented using 3D-Slicer, and radiomic features were extracted via Python. LASSO regression selected prognostic features for model integration. Clinicopathological data include tumour length, lymph node positivity, differentiation grade, and neurovascular infiltration. Ultimately, a machine learning model was established by combining the screened imaging feature data and clinicopathological data and validating model performance. A nomogram was constructed for survival prediction, and risk stratification was carried out through the prediction results of the machine learning model and the nomogram. Survival analysis was performed for stage-based patient subgroups across risk stratifications to identify adjuvant therapy-benefiting cohorts.

**Results:**

Patients were randomly divided into a 7:3 ratio of 368 patients in the training cohorts and 158 patients in the validation cohorts. The LASSO regression screens out 6 recurrence prediction and 9 recurrence pattern prediction features, respectively. Among 526 patients (mean age 63; 427 males), the model achieved high accuracy in predicting recurrence (training cohort AUC: 0.826 [logistic regression]/0.820 [SVM]; validation cohort: 0.830/0.825) and recurrence patterns (training:0.801/0.799; validation:0.806/0.798). Risk stratification based on a machine learning model and nomogram predictions revealed that adjuvant therapy significantly improved disease-free survival in stages II–III patients with predicted recurrence and low survival (HR 0.372, 95% CI: 0.206–0.669; *p* < 0.001).

**Conclusion:**

Machine learning models exhibit excellent performance in predicting recurrence after surgery for squamous oesophageal cancer.

**Critical relevance statement:**

Radiomic features of contrast-enhanced CT imaging can predict the prognosis of patients with oesophageal squamous cell carcinoma, which in turn can help clinicians stratify risk and screen out patient populations that could benefit from adjuvant therapy, thereby aiding medical decision-making.

**Key Points:**

- There is a lack of prognostic models for oesophageal squamous cell carcinoma in current research.
- The prognostic prediction model that we have developed has high accuracy by combining radiomics features and clinicopathologic data.
- This model aids in risk stratification of patients and aids clinical decision-making through predictive outcomes.

**Graphical Abstract:**

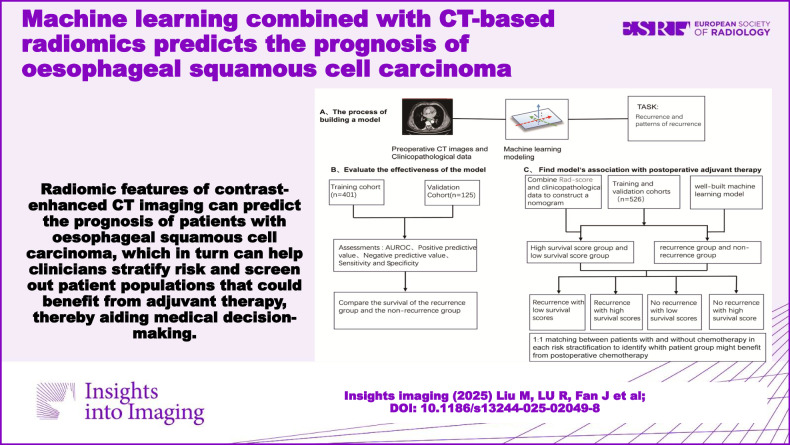

## Introduction

Oesophageal cancer is one of the most common malignancies worldwide and a leading cause of cancer-related deaths [1]. Despite advances in multimodal treatment of the disease, recurrence and mortality rates remain high [2, 3]. Patients with disease recurrence and metastasis have an extremely poor prognosis [4–6]. Therefore, predicting a patient’s risk of recurrence, including the potential site of recurrence, after radical surgery for oesophageal cancer is crucial. Such predictions can provide clinicians with valuable information for assessing patient prognosis, identifying those at high risk of postoperative recurrence, and developing individualised treatment plans to improve patient outcomes. Many scholars have developed nomogram models to predict prognosis, which have good performance, but cannot directly derive prognostic outcomes [7, 8]. At the same time, many studies have revealed more risk factors that affect the prognosis of oesophageal cancer after surgery, such as: the patterns of regional lymph node metastasis [9], biomarkers ACSL4 and GPX4 [10], preoperative laboratory data indicators [11], neutrophil-platelet score, and prognostic nutritional index [12]. These newly discovered prognostic predictors are instructive together with previously influencing factors such as pathological stage [13], tumour length and diameter [14], and neurovascular invasion [15]. Imaging plays a pivotal role in disease management by providing non-invasive visualisation of anatomical structures, functional dynamics, and pathological changes. Conventional modalities (e.g. CT, MRI, and PET) have revolutionised diagnosis, treatment planning, and therapeutic monitoring. However, the vast majority of imaging data remains underutilised, as human interpretation primarily focuses on qualitative or semi-quantitative features. Radiomics, a powerful method for extracting information from medical images, has demonstrated the ability to predict disease prognosis [16–20]. By extracting features from CT images and combining them with clinical and pathological data, radiomics can be integrated with machine learning techniques to provide more accurate predictions of patient prognosis. Most current studies have utilised radiomics to predict the degree of differentiation [21], neurovascular infiltration [22], and occult parapharyngeal lymph node metastases [23] in oesophageal cancer. We have developed a machine learning model that extracts information from preoperative CT images using radiomics and combines it with patients’ clinicopathological data to predict the recurrence and pattern of relapse in oesophageal cancer patients within 3 years postoperatively, providing clinicians with comprehensive prognostic information to identify patients who may benefit from postoperative adjuvant therapy.

## Methods

This single-centre retrospective study was approved by the Ethics Committees of The First Affiliated Hospital of Nanjing Medical University, and the requirement to obtain written informed consent was waived.

### Patient data collection

**Fig. 1:**
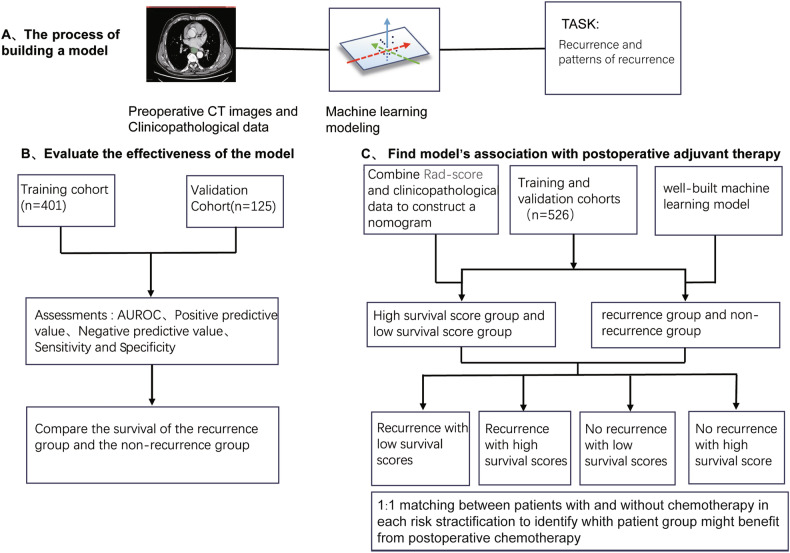
Study design for the development and validation of a machine learning model to predict recurrence and recurrence patterns. AUROC, area under the receiver operating characteristic; Rad-score, radiomics score

The overall research framework is illustrated in Fig. 1. From 25 May 2017 to 20 February 2021, consecutive patients who fulfilled the following inclusion criteria were identified: a confirmed diagnosis of squamous carcinoma of the lower and middle oesophagus, with the patient having undergone radical oesophageal cancer surgery; availability of a preoperative thoracic and upper abdominal enhanced CT scan; an ultrasound of the cervical lymph nodes to exclude cervical lymph node metastasis; and availability of detailed postoperative pathology reports and three-year postoperative follow-up data [24–26]. Exclusion criteria: inability of CT to identify the lesion; failure to achieve R0 resection. The study flow diagram can be found in Fig. S1 (Electronic Supplementary Material). The primary clinicopathological data collected included gender, age, most recent preoperative CT images, tumour length, lymph node positivity (lymph node metastasis/lymph node resection), degree of differentiation, percentage of eosinophils (eosinophils/total leucocytes), fibrinogen–albumin ratio, serum CEA levels (a glycoprotein biomarker often elevated in epithelial cancers), and patient follow-up for a minimum of three years [14, 15, 27–31]. All patients underwent a standard Ivor-Lewis or McKeown radical esophagectomy with a standard two-field lymph node dissection, including lymph nodes adjacent to the recurrent laryngeal nerve. The two procedures represent the standard treatment for oesophageal cancer, with outcomes that are comparable [32–34]. Additionally, preoperative cervical lymph node ultrasound and CT confirmed the absence of abnormal enlarged lymph nodes in all study participants. This is important because lower and middle oesophageal cancers with cervical lymph node metastasis are more likely to recur and may require cervico–thoracic–abdominal three-field lymph node dissection [35, 36], and the Japanese Oesophageal Society staging even classifies supraclavicular lymph node metastasis of lower oesophageal cancer as N3 [37, 38].

### Clinical outcome

Recurrence in patients was assessed using postoperative CT, cervical lymph node ultrasound, PET-CT, and puncture biopsy. Recurrence patterns are categorised into two types: (1) local regional recurrence, including anastomotic recurrence and regional lymph nodes recurrence. Regional lymph nodes mainly include cervical lymph nodes, mediastinal lymph nodes and 6 groups of lymph nodes in the abdominal cavity (15 groups of diaphragmatic lymph nodes, 16 groups of para-cardiac lymph nodes, 17 groups of left gastric artery lymph nodes, 18 groups of common hepatic artery lymph nodes, 19 groups of splenic artery lymph nodes, 20 groups of coeliac dry lymph nodes); and (2) distant metastatic recurrence, refers to the reappearance of cancer in organs or tissues far from the original tumour site after initial treatment. Disease-free survival is defined as the period from surgery to either tumour recurrence, death, or the last known instance of being free from recurrence. Overall survival is defined as the time from surgery to death from any cause. In this study, the primary endpoint was the time to tumour recurrence, while the secondary endpoint was overall survival.

### Development of machine learning models

**Fig. 2:**
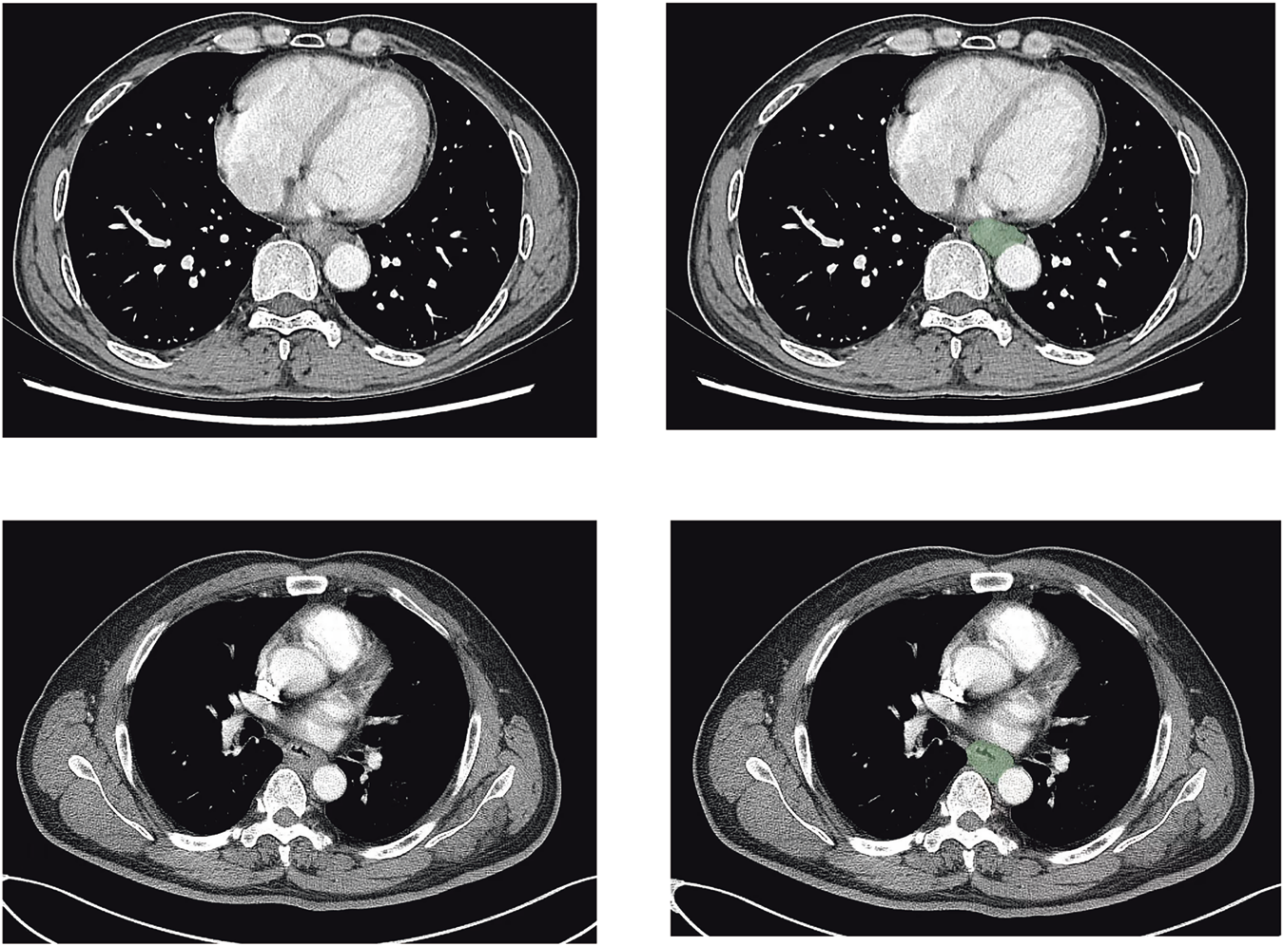
Appropriate CT images

The region of interest (ROI) for the tumour site in each patient’s CT image was mapped using 3D-Slice, generating a mask file co-imported with the original CT into Python (Fig. 2). PyRadiomics in Python extracted 1702 radiomic features, which were filtered to 302 via *t*-test. Least absolute shrinkage and selection operator (LASSO) regression (Fig. S2, Electronic Supplementary Material) with 10-fold cross-validation optimised regularisation parameter λ using the 1-SE criterion for model simplicity [39]. Six prognostic features and nine recurrence pattern features were identified (Appendix S1) (Electronic Supplementary Material). Clinicopathological parameters were selected via the Cox proportional hazards model before integrating with radiomic features into a Python-based machine learning model predicting 3-year recurrence and patterns. Synthetic minority over-sampling technique (SMOTE) addressed dataset imbalance during training. Feature selection and modelling codes are detailed in Appendix S2 (Electronic Supplementary Material).

### Evaluating model performance and the construction of a nomogram

Maximise sensitivity and specificity by selecting the optimal threshold based on the Youden Index within the training cohort. Assess metrics including the area under the receiver operating characteristic curve (AUC), sensitivity, specificity, and positive and negative predictive values. Additionally, a nomogram was constructed for individual survival assessment, which was used to stratify patients and identify those who would benefit from adjuvant therapy. Image features were obtained using the same screening method as described previously. Nine features crucial for individualised survival assessment were identified (Fig. S2, Electronic supplementary material). To evaluate the nomogram’s predictive value for prognosis, we generated calibration curves to compare predicted outcomes with actual outcomes and plotted decision curve analysis (DCA) to assess the model’s applicability in real clinical scenarios. The core code can be found in Appendix S2 (Electronic supplementary material).

### Clinical application of the models

After constructing the machine learning model and nomogram, we used the prediction results of these two models to stratify patients. Within each group, PSM was performed based on whether they received adjuvant therapy, and then screened out the patient population that could benefit from adjuvant therapy.

### Statistical analysis of data

Statistical analyses were conducted using SPSS (version 25.0), employing the χ² test and Fisher’s exact test for qualitative data, and the independent samples *t*-test for quantitative data. Cox proportional hazards regression models were also constructed with SPSS. Kaplan–Meier survival curves were generated and analysed using R (version 4.3.2), with statistical significance assessed via the log-rank test.

## Result

### Patient cohort and baseline characteristics

This retrospective study included 526 patients (average age, 63 years; 427 men). Among the patients, there were 427 males with an average age of 62.08. Within 3 years, 104 males relapsed, and 47 received neoadjuvant therapy before surgery. There were 99 females with an average age of 65; 21 females had recurrence within 3 years, and 7 had preoperative neoadjuvant treatment. Detailed clinicopathological characteristics of the patients are presented in Table 1. The five-year survival rate for these patients was 33.08%. There were 162 (30.6%) patients in stage I, 193 (36.7%) patients in stage II, and 171 (32.5%) patients in stage III. 54 (10.2%) patients received preoperative neoadjuvant therapy, while 267 (50.8%) received postoperative adjuvant therapy. Pathological factors such as lymph node positivity, degree of differentiation, and tumour length have been shown to be significant predictors of outcomes (Table S1) (Electronic supplementary material). Neurovascular infiltration was also included, as it has been shown to be predictive of outcomes.

**Table 1:** Characteristics of patients for the whole cohort according to recurrence

	All patients (*n* = 526)	Recurrence (*n* = 125)	No recurrence (*n* = 401)	*p*
Age	63 (58.00, 68.00)	63 (57.00, 68.00)	64 (58.00, 68.00)	0.495
Sex ratio (M:F)	427:99	104:21:00	323:78	
Differentiation status^a^				< 0.001
Well	53 (10.08)	3 (2.40)	50 (12.47)	
Moderate	270 (51.33)	52 (41.60)	218 (54.36)	
Poor and	203 (38.59)	70 (56.00)	133 (33.17)	
Undifferentiated				
TL&D	2.60 (1.92, 4.00)	3.50 (2.50, 4.50)	2.50 (1.50, 3.50)	< 0.001
CEA	2.35 (1.67, 3.41)	2.41 (1.77, 3.66)	2.35 (1.67, 3.33)	0.443
LVI^b^	125 (23.76)	44 (35.20)	81 (20.20)	< 0.001
EO% (%)	2.10 (1.34, 3.40)	2.10 (1.40, 3.20)	2.20 (1.30, 3.48)	0.714
LNPR (%)	0.00 (0.00, 0.07)	0.05 (0.00, 0.12)	0.00 (0.00, 0.05)	< 0.001
FAR	0.07 (0.06, 0.08)	0.07 (0.06, 0.08)	0.07 (0.06, 0.08)	0.344
ypT category^c^				< 0.001
ypT0	19 (3.61)	2 (1.60)	17 (4.24)	
ypT1	186 (35.36)	17 (13.60)	169 (42.14)	
ypT2	93 (17.68)	24 (19.20)	69 (17.21)	
ypT3	199 (37.83)	63 (50.40)	136 (33.92)	
ypT4	29 (5.51)	19 (15.20)	10 (2.49)	
NAT^d^				0.694
Yes	54 (10.27)	14 (11.20)	40 (9.98)	
No	472 (89.73)	111 (88.80)	361 (90.02)	
POAT^e^				< 0.001
Yes	267 (50.76)	82 (65.60)	185 (46.13)	
No	259 (49.24)	43 (34.40)	216 (53.87)	

Values in parentheses are median (25th percentile, 75th percentile) unless indicated otherwise

*TL&D* tumour length and diameter, *CEA* carcinoembryonic antigen, *LVI* lymphovascular invasion, *EO%* eosinophil percentage, *LNPR* lymph node positivity rate, *FAR* fibrinogen–albumin ratio, *NAT* neoadjuvant therapy, *POAT* postoperative adjuvant therapy

^a–e^ Values are percentages

### Recurrence patterns

Within 3 years post-surgery, 23.7% (125/526) of patients experienced recurrence, categorised as local 14 (1.1%), locoregional lymph node 26 (20.8%), cervical lymph node 21 (16.8%), or distant metastases 64 (51.2%).

### Predictive model performance

**Fig. 3:**
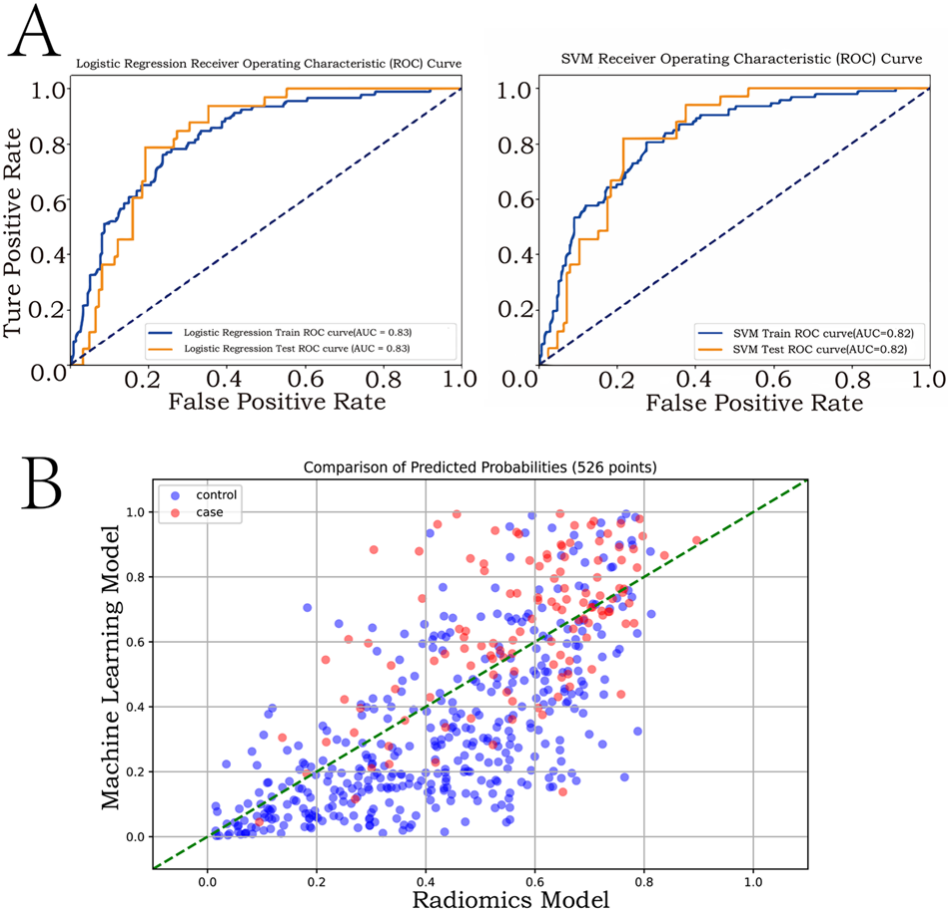
Model accuracy for predicting recurrence in the training and validation cohorts. **A** Both different algorithmic machine learning models predict recurrence with high accuracy. **B** The abscissa represents the probability of recurrence predicted by the radiomics model, and the ordinate represents the probability of recurrence predicted by the machine learning model. The blue dots represent non-relapsed individuals, and the red dots represent relapsed individuals. The truncated line represents an equal prediction probability for both models. For the machine learning model, most of the blue dots are less than 0.5 and distributed below the truncation line, and most of the red dots are more than 0.5 and distributed over the truncation line

Patients were randomly divided into a 7:3 ratio of 368 patients in the training cohorts and 158 patients in the validation cohorts. The logistic regression and support vector machines (SVM) models achieved robust discrimination in both training (AUC 0.826 and 0.820) and validation cohorts (AUC 0.830 and 0.825) (Fig. 3A and Table 2). Compared to radiomics-only (AUC 0.70) and clinicopathological-only models (AUC 0.65), the combined machine learning model demonstrated superior performance (net reclassification improvement [NRI] 0.127, 95% CI: 0.089–0.167; *p* < 0.001) (Fig. 3B) (Fig. S3, Electronic Supplementary Material).

**Table 2:** Performance of the machine learning model

	Sensitivity	Specificity	PPV	NPV
Logistic regression model				
Training cohort	91.1	73.9	50.0	91.0
Validation cohort	91.8	80.8	50.0	91.8
SVM				
Training cohort	91.3	68.8	46.2	91.3
Validation cohort	94.1	77.6	49.1	94.2

The models achieved high sensitivity (ability to correctly identify recurrence, > 91%) and negative predictive value (NPV; probability of true non-recurrence, > 91%) in both cohorts. Specificity (ability to correctly exclude non-recurrence) improved in validation cohorts (logistic regression: 80.8%; SVM: 77.6%). Positive predictive values (PPV; probability of true recurrence) remained modest (46.2–50.0%), likely due to class imbalance. This performance supports their utility for prioritising low-risk patients in postoperative surveillance

### Survival analysis and prediction of recurrence patterns

**Fig. 4:**
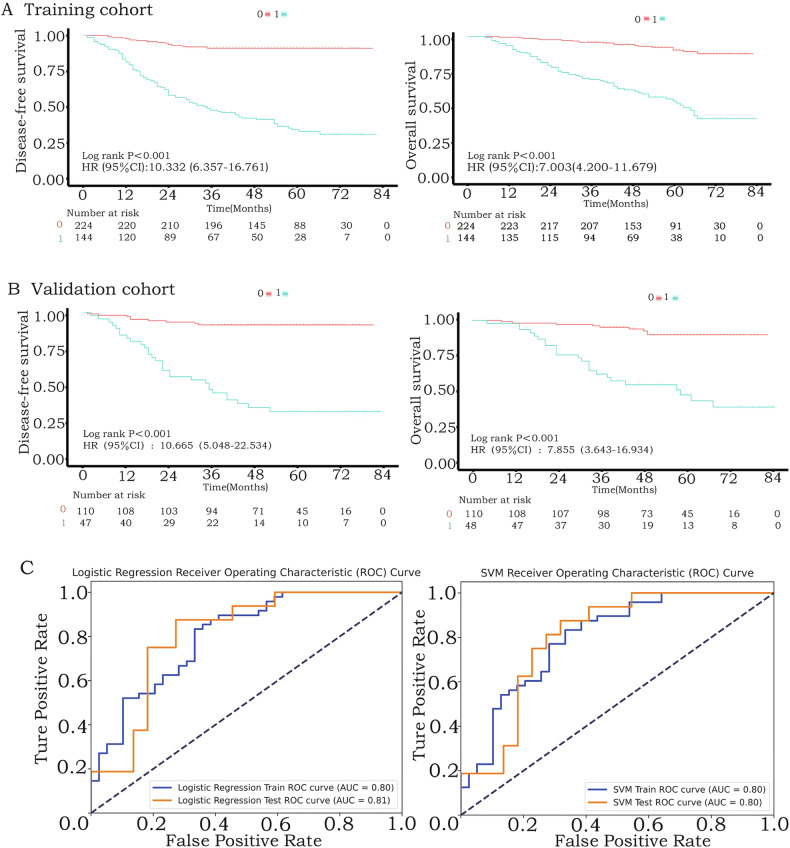
Survival analysis of predicted outcomes and accuracy in predicting recurrence patterns. **A** Disease-free survival and overall survival of the training cohort (*n* = 368), “0” represents the patient group with a predicted outcome of no recurrence. “1” represents the patient group with a predicted outcome of recurrence. **B** Disease-free survival and overall survival of validation cohort (*n* = 158). **C** The ROC curve for predicting recurrence patterns showed that the model had good performance

Kaplan–Meier analysis confirmed that patients predicted as non-recurrent by the model had significantly longer disease-free and overall survival (Fig. 4A, B). The model further stratified recurrence patterns (logistic regression AUC 0.80; SVM AUC 0.81), distinguishing local-regional from distant metastatic recurrence (Fig. 4C). Subgroup analyses across American Joint Committee on Cancer 8th edition T/N stages and differentiation grades revealed consistent prognostic value (Figs. S4–S6, Electronic supplementary material).

### Clinical translation: tool development and therapeutic implications

**Fig. 5:**
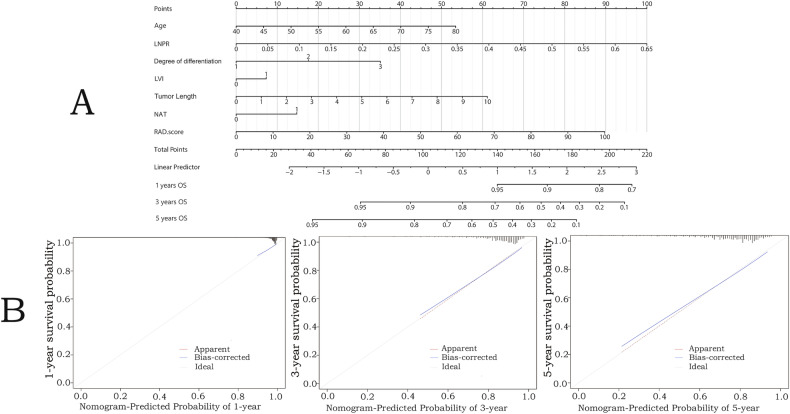
Developed nomogram and calibration curves for the nomogram. **A** Rad-score and clinicopathological data were used for building the nomogram. Draw a vertical line upward for each variable of the patient to obtain a single score, add up all the individual scores to obtain the total score, and draw a vertical line downward based on the total score to obtain the patient’s survival probability of 1 year, 3 years, and 5 years. **B** Calibration curves indicate the goodness of fit of the nomogram. The Ideal line represents the perfect match between the actual (*Y*­-axis) and nomogram-predicted (*X*-axis) probabilities (the 45° straight line). The closer the two curves are to the ideal curve, the higher the accuracy. The calibration plot revealed good predictive accuracy between the actual probability and predicted probability. LNPR, lymph node positivity rate; LVI, lymphovascular invasion; NAT, neoadjuvant therapy; Rad-score, radiomics score

**Fig. 6:**
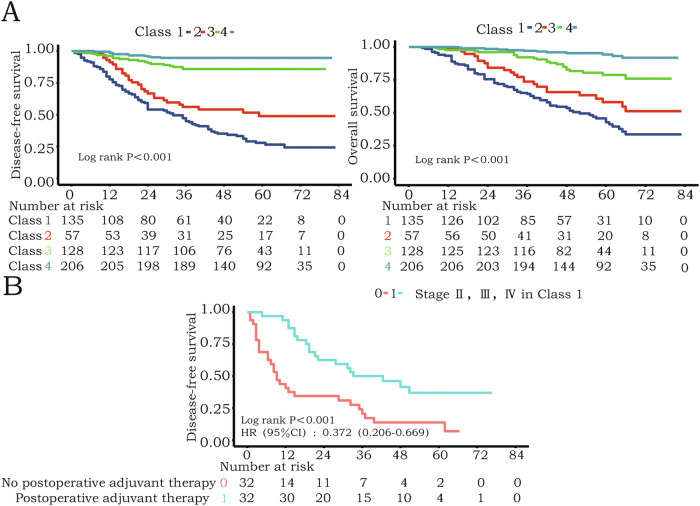
Further risk stratification of patients and their relationship with postoperative adjuvant therapy. Four different risk classes were defined by the machine learning model and Nomogram predictions. **A** Kaplan–Meier curve of disease-free survival and overall survival for patients who were stratified. **B** Kaplan–Meier curves of disease-free survival for patients who are stratified according to receipt of postoperative adjuvant therapy, patients with stage II, III, and IV in Class 1 (*n* = 64)

A nomogram integrating radiomic (Rad-score) and clinicopathological predictors demonstrated strong calibration with observed survival (Fig. 5A, B). Rad-score calculation and decision curve analysis are detailed in Appendix S3 and Fig. S7 (Electronic supplementary material). Additionally, we identified patient groups that would benefit from chemotherapy, with most adjuvant therapies being immune-chemotherapy treatments. Patients were first dichotomised into four groups based on the prediction results of the machine learning model and the nomogram scores (using the median). Table 3 presents examples of patients from each group and their survival outcomes. Significant differences in survival were observed among the four groups (Class 1 high recurrence and low survival, Class 2 high recurrence and high survival, Class 3 low recurrence and low survival, Class 4 low recurrence and high survival), suggesting that we have further stratified patients by risk successfully (Fig. 6A). Within each group, we performed 1:1 propensity score matching (PSM) to compare patients who received postoperative chemotherapy with those who did not, based on clinicopathological characteristics. After matching, patient characteristics were comparable between the two groups (Table 4). We found that for patients in Class 1 (high recurrence and low survival), postoperative chemotherapy was associated with improved disease-free survival in stages II, III (HR 0.372 [95% CI: 0.206–0.669], *p* < 0.001) (Fig. 6B), no similar correlations were observed in other patient stratifications (Fig. S8) (Electronic Supplementary Material).

**Table 3:** Example of patients from each group and their survival outcome

	Dercription	Outcomes
Class 1	A 52-year-old man did not undergo neoadjuvant therapy, and the postoperative pathology showed that the tumour was poorly differentiated, the tumour had a length of 5.8 cm, a positive rate of lymph nodes of 33.3%, and neurovascular invasion. Predicted results: recurrence within 3 years, 5-year survival rate 28.37% (Rad-score = 24.47)	DFS: 18 months OS: 37 months
Class 2	A 52-year-old woman who did not undergo neoadjuvant therapy. The pathological tumour was differentiated after surgery, the length and diameter of the tumour were 4 cm, the positive rate of lymph nodes was 10%, and there was no neurovascular invasion. Predicted outcome: recurrence within 3 years, 5-year survival rate of 80.04%. (Rad-score = 24.47)	DFS: 26 months OS: 52 months
Class 3	A 77-year-old male who did not receive preoperative neoadjuvant therapy showed poor tumour differentiation after surgery, with a positive lymph node rate of 16.1%, a tumour length diameter of 3.5 cm, and neurovascular invasion. Predicted outcome: no recurrence within 3 years. 5-year survival rate: 28.26%. (Rad-score = 24.40)	DFS: 42 months OS: 42 months
Class 4	A 54-year-old woman, did not receive preoperative neoadjuvant therapy. Pathology showed that the tumour was well differentiated, with a tumour diameter of 2 cm, negative neurovascular infiltration, and no lymph node metastasis. Predicted to be no recurrence within three years, the 5-year survival rate is 93.15% (Rad-score: 24.45)	Still alive after surgery at 56 months

**Table 4:** 1:1 PSM match in different patient stratifications according to whether postoperative adjuvant therapy

Stages II, III, and IV in Class 1 before PSM						
Variable	Total (*n* = 128)	0 (*n* = 32)	1 (*n* = 96)	Statistic	*p*	SMD
Age, M (Q₁, Q₃)	63.50 (59.00, 68.00)	67.00 (62.75, 71.25)	63.00 (57.00, 67.00)	*Z* = −2.918	0.004	−0.617
LNPR, M (Q₁, Q₃)	0.09 (0.04, 0.17)	0.09 (0.05, 0.14)	0.09 (0.04, 0.18)	*Z* = −0.083	0.934	0.024
TL, M (Q₁, Q₃)	4.00 (3.00, 4.73)	3.50 (2.50, 4.50)	4.00 (3.00, 4.85)	*Z* = −1.176	0.239	0.144
Degree of differentiation, *n* (%)				χ² = 0.244	0.621	
Moderate	28 (21.88)	8 (25.00)	20 (20.83)			−0.103
Poor	100 (78.12)	24 (75.00)	76 (79.17)			0.103
LVI, *n* (%)				χ² = 0.094	0.759	
No	63 (49.22)	15 (46.88)	48 (50.00)			0.062
Yes	65 (50.78)	17 (53.12)	48 (50.00)			−0.062

Stages II, III, and IV in Class 1 after PSM						
Variable	Total (*n* = 64)	0 (*n* = 32)	1 (*n* = 32)	Statistic	*p*	SMD
Age, M (Q₁, Q₃)	67.00 (61.75, 71.00)	67.00 (62.75, 71.25)	67.00 (60.00, 70.25)	*Z* = −0.484	0.628	−0.120
LNPR, M (Q₁, Q₃)	0.09 (0.00, 0.14)	0.09 (0.05, 0.14)	0.08 (0.00, 0.13)	*Z* = −1.003	0.316	−0.225
TL, M (Q₁, Q₃)	3.50 (2.50, 4.53)	3.50 (2.50, 4.50)	4.00 (3.22, 4.70)	*Z* = −0.708	0.479	0.045
Degree of differentiation, *n* (%)				χ² = 0.869	0.351	
Moderate	13 (20.31)	8 (25.00)	5 (15.62)			−0.258
Poor	51 (79.69)	24 (75.00)	27 (84.38)			0.258
LVI, *n* (%)				χ² = 0.250	0.617	
No	32 (50)	15 (46.88)	17 (53.12)			0.125
Yes	32 (50)	17 (53.12)	15 (46.88)			−0.125

Stage II in Classes 2 and 3 before PSM						
Variable	Total (*n* = 86)	0 (*n* = 35)	1 (*n* = 51)	Statistic	*p*	SMD
Age, M (Q₁, Q₃)	64.00 (58.00, 70.00)	66.00 (61.50, 70.50)	63.00 (57.50, 69.00)	*Z* = −2.073	0.038	−0.461
LNPR, M (Q₁, Q₃)	0.00 (0.00, 0.00)	0.00 (0.00, 0.00)	0.00 (0.00, 0.04)	*Z* = −1.749	0.080	0.218
TL, M (Q₁, Q₃)	3.00 (2.00, 4.15)	3.00 (2.00, 3.75)	3.20 (2.10, 4.50)	*Z* = −0.781	0.435	0.180
Degree of differentiation, *n* (%)				–	1.000	
Moderate	1 (1.16)	0 (0.00)	1 (1.96)			0.141
Poor	57 (66.28)	23 (65.71)	34 (66.67)			0.020
LVI, *n* (%)	28 (32.56)	12 (34.29)	16 (31.37)			−0.063
No				χ² = 0.020	0.887	
Yes	67 (77.91)	27 (77.14)	40 (78.43)			0.031
Age, M (Q₁, Q₃)	19 (22.09)	8 (22.86)	11 (21.57)			−0.031

Stage II in Classes 2 and 3 after PSM						
Variable	Total (*n* = 58)	0 (*n* = 29)	1 (*n* = 29)	Statistic	*p*	SMD
Age, M (Q₁, Q₃)	65.50 (58.25, 70.00)	65.00 (61.00, 69.00)	66.00 (58.00, 70.00)	*Z* = −0.281	0.779	−0.108
LNPR, M (Q₁, Q₃)	0.00 (0.00, 0.00)	0.00 (0.00, 0.00)	0.00 (0.00, 0.00)	*Z* = −0.673	0.501	0.002
TL, M (Q₁, Q₃)	3.10 (2.23, 4.42)	3.00 (2.20, 4.00)	3.50 (2.30, 4.50)	*Z* = −0.507	0.612	0.128
Degree of differentiation, *n* (%)				χ² = 0.377	0.539	
Moderate	44 (75.86)	21 (72.41)	23 (79.31)			0.170
Poor	14 (24.14)	8 (27.59)	6 (20.69)			−0.170
LVI, *n* (%)				χ² = 0.000	1.000	
No	46 (79.31)	23 (79.31)	23 (79.31)			0.000
Yes	12 (20.69)	6 (20.69)	6 (20.69)			0.000

Stages III and IV in Classes 2 and 3 before PSM						
Variable	Total (*n* = 65)	0 (*n* = 18)	1 (*n* = 47)	Statistic	*p*	SMD
Age, M (Q₁, Q₃)	63.00 (55.00, 68.00)	67.00 (61.00, 70.50)	62.00 (55.00, 67.00)	*Z* = −1.447	0.148	−0.396
LNPR, M (Q₁, Q₃)	0.07 (0.04, 0.10)	0.07 (0.06, 0.12)	0.07 (0.04, 0.09)	*Z* = −1.122	0.262	−0.569
TL, M (Q₁, Q₃)	3.50 (2.50, 4.20)	3.00 (2.50, 3.50)	3.50 (2.55, 4.50)	*Z* = −1.697	0.090	0.405
Degree of differentiation, *n* (%)				–	0.331	
Moderate	1 (1.54)	1 (5.56)	0 (0.00)			−0.461
Poor	38 (58.46)	11 (61.11)	27 (57.45)			−0.074
LVI, *n* (%)	26 (40)	6 (33.33)	20 (42.55)			0.186
No				χ² = 0.964	0.326	
Yes	37 (56.92)	12 (66.67)	25 (53.19)			−0.270
Age, M (Q₁, Q₃)	28 (43.08)	6 (33.33)	22 (46.81)			0.270

Stages III and IV in Classes 2 and 3 after PSM						
Variable	Total (*n* = 32)	0 (*n* = 16)	1 (*n* = 16)	Statistic	*p*	SMD
Age, M (Q₁, Q₃)	62.00 (55.00, 69.00)	64.50 (60.75, 69.50)	61.00 (54.00, 68.25)	*Z* = −0.982	0.326	−0.323
LNPR, M (Q₁, Q₃)	0.07 (0.04, 0.10)	0.07 (0.06, 0.11)	0.07 (0.04, 0.10)	*Z* = −0.830	0.407	−0.284
TL, M (Q₁, Q₃)	3.00 (2.50, 3.62)	3.00 (2.50, 3.50)	3.25 (2.88, 4.00)	*Z* = −0.671	0.502	0.059
Degree of differentiation, *n* (%)				–	1.000	
Moderate	21 (65.62)	10 (62.50)	11 (68.75)			0.135
Poor	11 (34.38)	6 (37.50)	5 (31.25)			−0.135
LVI, *n* (%)				–	1.000	
No	21 (65.62)	10 (62.50)	11 (68.75)			0.135
Yes	11 (34.38)	6 (37.50)	5 (31.25)			−0.135

Stages II, III, and IV in Class 4 before PSM						
Variable	Total (*n* = 85)	0 (*n* = 42)	1 (*n* = 43)	Statistic	*p*	SMD
Age, M (Q₁, Q₃)	62.00 (55.00, 65.00)	63.00 (59.00, 67.75)	57.00 (51.50, 63.00)	*Z* = −2.786	0.005	−0.619
LNPR, M (Q₁, Q₃)	0.00 (0.00, 0.03)	0.00 (0.00, 0.00)	0.00 (0.00, 0.04)	*Z* = −2.062	0.039	0.371
TL, M (Q₁, Q₃)	2.80 (2.00, 3.50)	3.00 (2.05, 3.50)	2.50 (1.75, 3.20)	*Z* = −0.957	0.339	−0.148
Degree of differentiation, *n* (%)				-	1.000	
Moderate	7 (8.24)	3 (7.14)	4 (9.30)			0.074
Poor	75 (88.24)	38 (90.48)	37 (86.05)			−0.128
LVI, *n* (%)	3 (3.53)	1 (2.38)	2 (4.65)			0.108
No				χ² = 0.113	0.736	
Yes	77 (90.59)	39 (92.86)	38 (88.37)			−0.140
Age, M (Q₁, Q₃)	8 (9.41)	3 (7.14)	5 (11.63)			0.140

Stages II, III, and IV in Class 4 after PSM						
Variable	Total (*n* = 60)	0 (*n* = 30)	1 (*n* = 30)	Statistic	*p*	SMD
Age, M (Q₁, Q₃)	61.00 (55.00, 65.00)	62.00 (56.00, 65.75)	61.00 (54.25, 65.00)	*Z* = −0.593	0.553	−0.162
LNPR, M (Q₁, Q₃)	0.00 (0.00, 0.00)	0.00 (0.00, 0.00)	0.00 (0.00, 0.00)	*Z* = −0.100	0.921	−0.104
TL, M (Q₁, Q₃)	2.65 (1.87, 3.35)	2.90 (1.85, 3.45)	2.50 (1.92, 3.20)	*Z* = −0.348	0.728	−0.042
Degree of differentiation, *n* (%)				–	1.000	
Moderate	4 (6.67)	2 (6.67)	2 (6.67)			0.000
Poor	54 (90)	27 (90.00)	27 (90.00)			0.000
LVI, *n* (%)	2 (3.33)	1 (3.33)	1 (3.33)			0.000
No				χ² = 0.000	1.000	
Yes	54 (90)	27 (90.00)	27 (90.00)			0.000
Age, M (Q₁, Q₃)	6 (10)	3 (10.00)	3 (10.00)			0.000

## Discussion

We developed and validated a machine learning model that integrates preoperative CT imaging with clinicopathological data to predict both recurrence and the pattern of recurrence up to three years after radical surgery for oesophageal cancer. This model has demonstrated advantages over radiomics models, producing consistent results across various clinical subgroups, highlighting its robustness. Clinically, this integration enables actionable risk stratification: high NPV (> 91%) permits reduced surveillance intensity for low-risk patients (e.g. fewer PET-CT scans), while recurrence pattern prediction (AUC 0.80–0.81) guides targeted imaging (e.g. prioritising endoscopic ultrasound for local-regional recurrence or PET-CT for distant metastases). Given that patients with similar clinical features can experience different outcomes, accurate prediction of recurrence is crucial for guiding decisions regarding postoperative adjuvant therapy. At the same time, we further used the prediction results of the model, combined with the prediction results of the constructed nomogram model, to stratify the risk of patients and screen the patient groups who benefited from postoperative adjuvant therapy. This facilitates the establishment of an individualised treatment plan.

It is important to note that all patients underwent preoperative cervical lymph node ultrasound to exclude cervical lymph node metastasis, and no clearly enlarged cervical lymph nodes were observed on enhanced CT scans. In contrast, immediate surgical intervention in patients with preoperative cervical lymph node metastasis may not be optimal; such cases should instead be managed with neoadjuvant therapy to evaluate the efficacy of subsequent surgery. Most of our neoadjuvant patients receive chemoimmunotherapy for two cycles. Numerous studies have demonstrated that preoperative chemoimmunotherapy is associated with high pathological complete response (PCR) and R0 resection rates, as well as a favourable safety profile in oesophageal squamous cell carcinoma [40–43]. Postoperative adjuvant therapy or treatment for inoperable advanced oesophageal cancer can also be based on chemoimmunotherapy, which significantly improves patient survival [44, 45].

Predicting the recurrence of oesophageal cancer after surgery is a challenging task. Rahman et al used easy-to-collect clinical data to build a machine learning model to predict the recurrence of oesophageal adenocarcinoma within 1 year (AUC 0.805) [46]. Although their model demonstrates the value of machine learning for clinical endpoints, it lacks the spatial heterogeneity information captured by radiomics. In recent radiomics studies, such as Tong [19] and Peng [18] et al, a nomogram was constructed to predict the survival rate of oesophageal squamous cell carcinoma after surgery using radiomics features and clinicopathological data. Although their model performed well, there was a lack of prediction of clinical outcomes such as recurrence or site of recurrence.

When constructing the machine learning model, we addressed the imbalance between recurrence and non-recurrence datasets by applying SMOTE to generate synthetic samples and balance the class distribution. This approach enhances the model’s ability to identify relapsed patients and mitigates overfitting. However, it also increases computational complexity.

Postoperative adjuvant therapy is a standard treatment for locally advanced oesophageal squamous cell carcinoma following surgery. However, the selection of suitable patients is largely based on clinicians’ empirical judgement, and there are no established criteria. In this study, patients were risk-stratified by integrating recurrence data with survival scores to identify those who might benefit from adjuvant therapy. The survival analysis found that patients with stages II and III in Class 1 did benefit from postoperative adjuvant therapy.

This study has several limitations. First, it is a single-centre clinical trial with a relatively homogeneous patient population primarily from East Asia; patients from other regions may exhibit different clinical characteristics and pathological subtypes. Second, despite including a substantial number of patients, the study’s retrospective nature introduces fixation bias and confounding factors. Third, while the developed model performs adequately in predicting recurrence in patients with medium-stage tumours, its performance may decline in identifying recurrences in early-stage tumours (T1–2N0) as the number of such patients increases. Finally, when screening patients who benefited from chemotherapy, no significant difference in survival was observed between the two groups of patients (with or without postoperative adjuvant therapy) in Class 2, Class 3, and Class 4. This lack of difference may be attributed to the small sample size within each stage group after PSM. Future work should prioritise multi-centre clinical modelling and assess the generalizability of the model across diverse patient populations. Emphasis should be placed on enhancing recurrence risk identification in early-stage patients. Regarding the model, we did not refine the prediction of recurrence sites (e.g. subdividing local regional recurrence into anastomotic recurrence and regional lymph node recurrence) because the model consistently underperformed due to insufficient recurrence cases. In the future, we plan to enhance the model’s capability in this area.

In conclusion, we have developed a stable and accurate machine learning model using preoperative CT images and clinicopathological data to predict recurrence and recurrence patterns in patients with oesophageal squamous cell carcinoma up to 3 years post-surgery. By outperforming radiomics-only models and enabling recurrence subtype prediction, our framework addresses a critical gap in ESCC management. These results can be utilised in prospective studies to evaluate their clinical utility and guide personalised medicine.

## Supplementary information

ELECTRONIC SUPPLEMENTARY MATERIAL

## Data Availability

The data that support the findings of this study are available from the corresponding authors with a signed data access agreement. De-identified patient-level clinical and outcome data will be provided upon reasonable request. The CT image data are not publicly available because they contain sensitive information that could compromise patient privacy. The source code for the machine learning model is available in the Electronic Supplementary Material.

## Abbreviations

AUC: Area under the receiver operating characteristic curve
LASSO: Least absolute shrinkage and selection operator
PSM: Propensity score matching
SMOTE: Synthetic minority over-sampling technique
SVM: Support vector machines
