# Supplementary material for: Machine learning combined with CT-based radiomics predicts the prognosis of oesophageal squamous cell carcinoma
Source: Insights Imaging. 2025 Oct 1;16:211. doi: 10.1186/s13244-025-02049-8 (PMC12488548; doi:10.1186/s13244-025-02049-8)

Machine learning combined with CT-based radiomics predicts the prognosis of oesophageal squamous cell carcinoma

ELECTRONIC SUPPLEMENTARY MATERIAL

Supplementary Appendixes S1-S3--page2-page13
Figure S1-S2---page14,15
Table S1---page16
Figure S3-S7---page16-page20
Figure S8---page21

Supplementary Appendixes S1

Feature selection

LASSO is a statistical method for feature selection and regression analysis. It implements feature selection by adding an L1 regularisation term to the objective function, driving some of the coefficients to zero and thus making the model sparser. In a linear regression problem, LASSO minimises the following objective function:

$$\text{minimize}(1/2m)||y - X\beta||^2_2 + \lambda||\beta||_1$$

y is the dependent variable, X is the matrix of independent variables, β is the vector of coefficients of the model representing the weights of each feature, m is the sample size, $||\text{-}||_2$ denotes the number of L2 paradigms, $||\text{-}||_1$ denotes the number of L1 paradigms, and λ is the regularisation parameter.

LASSO balances the goodness-of-fit and the sparsity of the model by controlling the size of λ. Larger values of λ push certain coefficients to zero more strongly, enabling feature selection; smaller values of λ focus more on fitting the data.

Below are the steps for tuning the regularisation parameter λ and selecting the optimal prognostic predictive features using 10-fold cross-validation:

1. initialise a range of λ values to be tuned, setting the range on a logarithmic scale: λ = [0.001, 0.01, 0.1, 1, 10, 100].
2. For each λ value, perform 10-fold cross-validation:

a. Divide the dataset into 10 subsets.

b. For each subset, use it as the validation set and the other 9 subsets as the training set.

c. Train using the LASSO regression model and calculate the prediction error (e.g., root-mean-square error) as a model performance metric.

d. Repeat steps b and c until all subsets are used as validation sets.

e. Calculate the average performance metric (e.g., average root-mean-square error) as the model performance at that value of λ.

3. Select the best λ value based on the average performance metric. Usually the value of λ with the smallest average performance metric is chosen because smaller mean error means better model performance.
4. retrain the LASSO model with the complete training set under the selected optimal λ and obtain the corresponding coefficient vectors.
5. filter the important prognostic predictive features based on the coefficient vectors. Features with zero coefficients can be considered irrelevant or unimportant.

This allows for the selection of the best regularisation parameter λ by 10-fold cross-validation, as well as the screening of optimal prognostic prediction features using the LASSO regression algorithm.

These feature names correspond to different types of image features extracted from medical imaging data. They are commonly used in radiomics analysis to extract quantitative information from images to assist in disease diagnosis, treatment evaluation, etc.

The following six are the features used in machine modeling

1.original_shape_LeastAxisLength: This is a shape feature that represents the length of the shortest axis of the target (e.g., tumour) in the image. It reflects the shape and size information of the target.

2.log-sigma-1-0-mm-3D_firstorder_90Percentile: This is a three-dimensional first-order statistical feature extracted after logarithmic scale space filtering (sigma=1.0 mm), representing the 90th percentile of the grayscale values in the image. Logarithmic scale space filtering enhances image details at different scales.

3.wavelet-LLH_glcm_Idn: This is a texture feature extracted using wavelet transform (low-frequency, low-frequency, high-frequency subbands) and gray-level co-occurrence matrix (GLCM), known as "Inverse Difference Normalized" (IDN), which reflects the contrast and smoothness of the image texture.

4.wavelet-HLH_gcm_Idn: This is a texture feature extracted using wavelet transform (high-frequency, low-frequency, high-frequency subbands) and gray-level co-occurrence matrix, also representing contrast and smoothness of the image texture.

5.log-sigma-1-0-mm-3D_glcm_Idn: This is a texture feature extracted after logarithmic scale space filtering, using gray-level co-occurrence matrix， IDN.

6.log-sigma-1-0-mm-3D_glcm_MaximumProbability: This is a texture feature extracted after logarithmic scale space filtering, using gray-level co-occurrence matrix, representing the probability value of the grayscale pair with the maximum probability in the co-occurrence matrix.

The following nine are the features used in predicting recurrence patterns

1.original_shape_Elongation:Measures how elongated a shape is compared to a perfect sphere. It's a ratio that quantifies the extent to which a shape deviates from being spherical. Used to assess the shape of structures in an image, which can be indicative of certain conditions or anomalies.

2.original_shape_SurfaceArea: The total surface area of a shape in the image. Provides information about the size and the surface complexity of the structure being analyzed.

3.log-sigma-1-0-mm-3D_firstorder_Skewness: Skewness is a measure of the asymmetry of the intensity distribution of the image at a specific scale (in this case, 1.0 mm). The "log-sigma" indicates that the feature was computed from the image after applying a log transformation to the intensities. Helps in understanding the distribution of intensity values within the image, which can be useful for distinguishing different tissue types or detecting abnormalities.

4.log-sigma-3-0-mm-3D_firstorder_90Percentile: This feature represents the 90th percentile of the intensity values in the image at a scale of 3.0 mm after applying a log transformation. Provide information about the intensity distribution, particularly the high-end values, which can help in distinguishing between different structures or abnormalities.

5.wavelet-LLH_firstorder_Mean: Mean of the intensity values after applying a wavelet transform with specific filter settings (LLH: Low-Low-High). Helps in capturing texture and structural features by analyzing the image at different scales and orientations.

6.wavelet-LLH_firstorder_Skewness: Skewness of the intensity values after wavelet transformation with LLH settings. Provides insight into the asymmetry of texture features at different scales, which can be useful for texture classification and analysis.

7.wavelet-LLH_glcm_ClusterShade: Cluster Shade is a measure derived from the Gray Level Co-occurrence Matrix (GLCM) after wavelet transformation with LLH settings. It assesses the shading of clusters of co-occurring pixel values. Be used to describe the spatial arrangement and distribution of intensity values, which helps in texture analysis.

8.wavelet-LLH_glcm_InverseVariance: Inverse Variance is a GLCM feature that measures the contrast and variability of pixel values after wavelet transformation with LLH settings. Provides information about the variability of texture patterns, which can be useful for distinguishing different tissue types or detecting anomalies.

9.wavelet-LHH_glcm_Imc1:IMC1 (Information Measure of Correlation 1) is a GLCM feature that measures the correlation between different intensity levels in the image after wavelet transformation with LHH settings. Helps in understanding the statistical dependency between pixel values, which is important for texture classification and analysis.

The following nine are the features used in nomogram

1.original_shape_Flatness: Flatness of the shape. Describes how flat the shape of an area is. Typically used to assess the regularity or irregularity of a shape.

2. original_shape_LeastAxisLength: The minimum axis length of the shape. Indicates the minimum major axis length of the contour, a value commonly used to describe the size of a shape.

3. 10g.sigma.1.0.mm.3D firstorder_9aPercentile: The 90th percentile of the grey levels of the 3D image at a scale of 1.0 mm. The distribution of grey values in the image is described, especially for high grey values.

4. log·sigma.3.0.mm.3D_glrlm Runvariance: The running variance of the greyscale run length matrix (GLRM) of a 3D image at a scale of 3.0 mm. Reflects the degree of variation in the run length of grey values in the image, which may involve texture features.

5. 1og.sigma.3.0.mm.3D_glszm LargeAreaHighGrayLevelEmphasis: High grey level emphasis of large regions of the grey size region matrix (GLSZM) of a 3D image at a scale of 3.0 mm. Describes the intensity of the high grey level of large regions in the image.

6. wavelet.LLH firstorder_kurtosis: Kurtosis of the grey scale distribution in the LLH (low-low-high) subband after wavelet decomposition. The inverse 0 maps the thickness of the tails of the distribution and is often used to assess the complexity of the texture.

7. wavelet.LHL firstorder_mean: The mean of the grey level distribution in the LHL (low-high-low) subband after wavelet decomposition. Describes the average level of grey values in this subband.

8. wavelet.HHL_glszm sizeZoneNonuniformityNormalized: Non-uniformity of normalised size regions in the HHL (High-High-Low) subband after wavelet decomposition. Describes the grey level non-uniformity of different size regions in the image.

9. wavelet.HHH_firstorder Median: The median of the grey scale distribution in the HHH (High-High-High) subband after wavelet decomposition.The median is the location of the centre of the grey values and is often used to describe the central tendency of the distribution.

Supplementary Appendixes S2

Feature selection(python):

```
import …
para_path = 'yaml/1.yaml'
extractor = featureextractor.RadiomicsFeatureExtractor(para_path)
data_A = pd.DataFrame()
list_1 = np.arange(1,126) #
dir = 'data/MyData/'
for i in list_1:
    imgname = dir + 'FF' + '/' + 'FF' + str(i) + 'RAW.nrrd'
    maskname = dir + 'FF' + '/' + 'FF' + str(i) + 'MASK.nrrd'
    featureVector = extractor.execute(imgname,maskname)
    df_add = pd.DataFrame([featureVector])
    data_A = pd.concat([data_A, df_add], ignore_index=True)
    print(i)
data_A.to_excel('excel/FF.xlsx', index=False)
data_B = pd.DataFrame()
list_2 = np.arange(1,402)#
for i in list_2:
    imgname = dir + 'WFF' + '/' + 'WFF' + str(i) + 'RAW.nrrd'
    maskname = dir + 'WFF' + '/' + 'WFF' + str(i) + 'MASK.nrrd'
    featureVector = extractor.execute(imgname,maskname)
    df_add = pd.DataFrame([featureVector])
    data_B = pd.concat([data_B, df_add], ignore_index=True)
    print(i)
data_B.to_excel('excel/WFF.xlsx', index=False)
```

t test(python):

```
import …
classinformation = tData["label"].unique()
df_dict = {}
for temp_classinformation in classinformation:
    df_dict[temp_classinformation] = tData[tData['label'] == temp_classinformation]
df0 = df_dict[classinformation[0]]
df1 = df_dict[classinformation[1]]
columns_index = []
for column_name in tData.columns[2:]:
    if levene(df1[column_name], df0[column_name])[1] > 0.05:
        if ttest_ind(df1[column_name], df0[column_name], equal_var=True)[1] < 0.05:
            columns_index.append(column_name)
    else:
        if ttest_ind(df1[column_name], df0[column_name], equal_var=False)[1] < 0.05:
            columns_index.append(column_name)
```

Lasso regression(R):

```
tData <- read.csv("./data.csv", fileEncoding = "UTF-8-BOM")
dim(tData)
colSums(is.na(tData))
set.seed(1234)
tData$status <- as.factor(tData$status)
trainIndex <- sample(1:nrow(tData), 0.8 * nrow(tData))
train <- tData[trainIndex,]
test <- tData[-trainIndex,]
dim(train)
dim(test)
library(glmnet)
x <- as.matrix(train[, c(-1, -2)])
dim(train)
str(x)
y <- as.numeric(train$status)
colMeans(x)
apply(x, 2, sd)
x <- scale(x, center = TRUE, scale = TRUE)
la_md <- glmnet(x, y, lambda = 0.1,
            family = 'binomial',
            alpha = 1)
la.md <- glmnet(x, y, family = "binomial", alpha = 1)
mod_cv <- cv.glmnet(x, y, alpha = 1, family = "binomial")
pdf("Cross_Validation.pdf")
plot(mod_cv)
abline(v = log(c(mod_cv$lambda.min, mod_cv$lambda.1se)), lty = 2)
dev.off()
lambda <- mod_cv$lambda.min
Coefficients <- coef(la.md, s = lambda)
Active.Index <- which(Coefficients != 0)
Active.Coefficients <- Coefficients[Active.Index]
Active.Index
Active.Coefficients
row.names(Coefficients)[Active.Index]
pdf("Lasso_Dynamic_Process.pdf")
plot(la.md, xvar = "lambda", label = TRUE)
abline(v = log(lambda), lty = 2)
dev.off()
print(paste(mod_cv$lambda.min, log(mod_cv$lambda.min)))
print(paste(mod_cv$lambda.1se, log(mod_cv$lambda.1se)))
best_lambda <- mod_cv$lambda.min
best_lambda
```

Machine learning modeling and model performance evaluation(python):

```
import …
file_path = 'excel/data.xlsx'
df = pd.read_excel(file_path, header=0)
df = df[['name', 'LNPR', 'Degree of differentiation', 'LVI', 'Tumor length', 'NAT',
        …… 'label']]

# Extract features and target variable
X = df[['LNPR', 'Degree of differentiation', 'LVI', 'Tumor length', 'NAT',
      ……]]  # Features
y = df['label']  # Target
names = df['name']
X_train, X_test, y_train, y_test, train_names, test_names = train_test_split(X, y, names,
test_size=0.3,random_state=50)
scaler = StandardScaler()
X_train_scaled = scaler.fit_transform(X_train)
X_test_scaled = scaler.transform(X_test)
smote = SMOTE(random_state=42)
X_train_resampled, y_train_resampled = smote.fit_resample(X_train_scaled, y_train)
models = {
    'Logistic Regression': {
        'model': LogisticRegression(class_weight='balanced', max_iter=500),
        'params': {}
    },
    'SVM': {
        'model': SVC(kernel='linear', class_weight='balanced', probability=True),
        'params': {
            'C': [0.1, 1, 10, 100]
        }
    }
}

train_predictions = pd.DataFrame(index=train_names)
test_predictions = pd.DataFrame(index=test_names)
results = {}
for name, model_info in models.items():
    model = model_info['model']
    params = model_info['params']

    if params:
        # Perform grid search to find the best parameters
        grid_search = GridSearchCV(model, params,
cv=RepeatedStratifiedKFold(n_splits=10, n_repeats=3, random_state=42),
                                   scoring='roc_auc')
        grid_search.fit(X_train_resampled, y_train_resampled)
        model = grid_search.best_estimator_
        print(f"Best parameters for {name}: {grid_search.best_params_}")
    cv = RepeatedStratifiedKFold(n_splits=10, n_repeats=3, random_state=42)
    cv_scores = cross_val_score(model, X_train_resampled, y_train_resampled, cv=cv,
```

```
scoring='roc_auc')
    mean_auc = np.mean(cv_scores)
    std_auc = np.std(cv_scores)
    ci_auc = (mean_auc - 1.96 * std_auc, mean_auc + 1.96 * std_auc)
    print(f"{name} Cross-validation AUC scores: {cv_scores}")
    print(f"{name} Mean cross-validation AUC score: {mean_auc:.4f}")
    print(f"{name} 95% Confidence Interval for AUC: {ci_auc}")
    model.fit(X_train_resampled, y_train_resampled)
    y_train_pred = model.predict(X_train_scaled)
    y_train_prob = model.predict_proba(X_train_scaled)[:, 1]
    fpr_train, tpr_train, _ = roc_curve(y_train, y_train_prob)
    roc_auc_train = auc(fpr_train, tpr_train)
    accuracy_train = accuracy_score(y_train, y_train_pred)
    precision_train = precision_score(y_train, y_train_pred)
    recall_train = recall_score(y_train, y_train_pred)
    f1_train = f1_score(y_train, y_train_pred)
    cm_train = confusion_matrix(y_train, y_train_pred)
    tn_train, fp_train, fn_train, tp_train = cm_train.ravel()
    specificity_train = tn_train / (tn_train + fp_train)
    ppv_train = tp_train / (tp_train + fp_train)
    npv_train = tn_train / (tn_train + fn_train)
    y_test_pred = model.predict(X_test_scaled)
    y_test_prob = model.predict_proba(X_test_scaled)[:, 1]
    fpr_test, tpr_test, _ = roc_curve(y_test, y_test_prob)
    roc_auc_test = auc(fpr_test, tpr_test)
    accuracy_test = accuracy_score(y_test, y_test_pred)
    precision_test = precision_score(y_test, y_test_pred)
    recall_test = recall_score(y_test, y_test_pred)
    f1_test = f1_score(y_test, y_test_pred)
    cm_test = confusion_matrix(y_test, y_test_pred)
    tn_test, fp_test, fn_test, tp_test = cm_test.ravel()
    specificity_test = tn_test / (tn_test + fp_test)
    ppv_test = tp_test / (tp_test + fp_test)
    npv_test = tn_test / (tn_test + fn_test)
    if name == 'Logistic Regression':
        coefficients = model.coef_[0]
        intercept = model.intercept_[0]
        feature_names = X.columns.tolist()
        equation = f"{name} Equation: "
        for feature, coef in zip(feature_names, coefficients):
            equation += f"({coef:.4f} * {feature}) + "
        equation += f"{intercept:.4f}"
        print(equation)
    results[name] = {
        'Train AUC': roc_auc_train,
        'Test AUC': roc_auc_test,
        'Train Accuracy': accuracy_train,
        'Test Accuracy': accuracy_test,
        'Train Precision': precision_train,
        'Test Precision': precision_test,
```

```
        'Train Recall': recall_train,
        'Test Recall': recall_test,
        'Train F1-score': f1_train,
        'Test F1-score': f1_test,
        'Train Specificity': specificity_train,
        'Test Specificity': specificity_test,
        'Train PPV': ppv_train,
        'Test PPV': ppv_test,
        'Train NPV': npv_train,
        'Test NPV': npv_test,
        'Train Confusion Matrix': cm_train,
        'Test Confusion Matrix': cm_test,
        'Confidence Interval for AUC': ci_auc
    }
    train_predictions[name + ' Prediction'] = y_train_pred
    test_predictions[name + ' Prediction'] = y_test_pred
    plt.figure()
    plt.plot(fpr_train, tpr_train, color='blue', lw=2, label=f'{name} Train ROC curve (AUC =
{roc_auc_train:.2f})')
    plt.plot(fpr_test, tpr_test, color='darkorange', lw=2, label=f'{name} Test ROC curve
(AUC = {roc_auc_test:.2f})')
    plt.plot([0, 1], [0, 1], color='navy', lw=2, linestyle='--')
    plt.xlim([0.0, 1.0])
    plt.ylim([0.0, 1.05])
    plt.xlabel('False Positive Rate')
    plt.ylabel('True Positive Rate')
    plt.title(f'{name} Receiver Operating Characteristic (ROC) Curve')
    plt.legend(loc="lower right")
    plt.show()
train_df = pd.concat(
    [train_names.reset_index(drop=True),
pd.DataFrame(X_train).reset_index(drop=True), y_train.reset_index(drop=True),
train_predictions.reset_index(drop=True)],
    axis=1)
test_df = pd.concat(
    [test_names.reset_index(drop=True), pd.DataFrame(X_test).reset_index(drop=True),
y_test.reset_index(drop=True), test_predictions.reset_index(drop=True)],
    axis=1)
train_df.to_excel('train_data_with_predictions.xlsx', index=False)
test_df.to_excel('test_data_with_predictions.xlsx', index=False)
```

Nomogram(R):

```
tData_train <- read.csv("./cox1.csv", fileEncoding = "UTF-8-BOM")
dim(tData_train)
str(tData_train)
dd <- datadist(tData_train)
options(datadist = "dd")
coxfit_raw <- cph(Surv(OS, status) ~ age + LNPR + Degree.of.differentiation + LVI +
Tumor.length + NAT + ……,data = tData_train, x = TRUE, y = TRUE, surv = TRUE)
coefficients_raw <- coef(coxfit_raw)
image_features <- c(……)
tData_train$RAD.score <- rowSums(tData_train[image_features] *
coefficients_raw[image_features])
rad_score_min <- min(tData_train$RAD.score, na.rm = TRUE)
rad_score_max <- max(tData_train$RAD.score, na.rm = TRUE)
tData_train$RAD.score <- (tData_train$RAD.score - rad_score_min) / (rad_score_max -
rad_score_min) * 100
tData_train <- tData_train[, !names(tData_train) %in% image_features]
coxfit_updated <- cph(Surv(OS, status) ~ age + LNPR + Degree.of.differentiation + LVI +
Tumor.length + NAT + RAD.score,
                  data = tData_train, x = TRUE, y = TRUE, surv = TRUE)
dd <- datadist(tData_train)
options(datadist = "dd")
surv <- Survival(coxfit_updated)
surv1 <- function(x) surv(12, x)
surv2 <- function(x) surv(12*3, x)
surv3 <- function(x) surv(12*5, x)
nom <- nomogram(coxfit_updated,
             fun = list(surv1, surv2, surv3),
             lp = TRUE,
             funlabel = c('1-year survival Probability',
                         '3-year survival Probability',
                         '5-year survival Probability'),
             maxscale = 100,
             fun.at = c(0.95, 0.9, 0.8, 0.7, 0.6, 0.5, 0.4, 0.3, 0.2, 0.1))
plot(nom,
    lplabel="Linear Predictor",
    xfrac = 0.2,
    tcl = -0.2,
    lmgp = 0.1,
    points.label = 'Points',
    total.points.label = 'Total Points',
    cap.labels = FALSE,
    cex.var = 1,
    cex.axis = 1,
    col.grid = gray(c(0.8, 0.95)))
```

Calibration curve and decision curve for nomogram(R):

```
cal1<-calibrate(coxfit_updated1,method='boot',u=12,m=200,B=1000)
 plot(cal1,lwd=1,lty=2,
     errbar.col=c(rgb(0,118,192,maxColorValue = 255)),
     xlab='Nomogram-Predicted Probability of 1-year',
     ylab='1-year survival probability',
     col=c(rgb(192,98,83,maxColorValue = 255)),
          subtitles=FALSE,
     xlim = c(0,1),ylim = c(0,1))
coxfit_updated2 <- cph(Surv(OS, status) ~ age + LNPR + Degree.of.differentiation + LVI
+ Tumor.length + NAT + RAD.score,
                    data = tData_train, x = TRUE, y = TRUE, surv = TRUE,time.inc = 36)
cal2<-calibrate(coxfit_updated2,method='boot',u=36,m=200,B=1000)
plot(cal2,lwd=1,lty=2,
     errbar.col=c(rgb(0,118,192,maxColorValue = 255)),
     xlab='Nomogram-Predicted Probability of 3-year',
     ylab='3-year survival probability',
     col=c(rgb(192,98,83,maxColorValue = 255)),
          subtitles=FALSE,
     xlim = c(0,1),ylim = c(0,1))
coxfit_updated3 <- cph(Surv(OS, status) ~ age + LNPR + Degree.of.differentiation + LVI
+ Tumor.length + NAT + RAD.score,
                    data = tData_train, x = TRUE, y = TRUE, surv = TRUE,time.inc = 60)
cal3<-calibrate(coxfit_updated3,method='boot',u=60,m=200,B=1000)
plot(cal3,lwd=1,lty=2,
     errbar.col=c(rgb(0,118,192,maxColorValue = 255)),
     xlab='Nomogram-Predicted Probability of 5-year',
     ylab='5-year survival probability',
     col=c(rgb(192,98,83,maxColorValue = 255)),
          subtitles=FALSE,
     xlim = c(0,1),ylim = c(0,1))
dca_results <- decision_curve(Surv(OS,status) ~ prob1, data = tData_train)
dca_results <- decision_curve(Surv(OS, status) ~ lin_pred, data = tData_train,
                          thresholds = seq(0, 1, by = 0.01))
tData_train$prob1 <- c(1-(summary(survfit(coxfit_updated1, newdata=tData_train),
times=12)$surv))
dca1 <- dcurves::dca(Surv(months,status) ~ prob1,
                   data = train,
                   time = 12,)
  dcurves::as_tibble()
ggplot(dca1,aes(x=threshold, y=net_benefit,color=variable))+
  stat_smooth(method = "loess", se = FALSE, formula = "y ~ x", span = 0.2) +
  coord_cartesian(ylim = c(0, 0.2)) +
  scale_x_continuous(labels = scales::label_percent(accuracy = 1)) +
  labs(x = "Risk Threshold", y = "Net Benefit", color = "") +
  theme_bw()
```

Supplementary Appendixes S3

Coefficient scaled:

age 0.238977178
LNPR 0.378147360
Degree of differentiation 0.314012697
LVI 0.087613516
Tumor length 0.250888689
NAT 0.110958578
original shape Flatness 0.039109537
original shape LeastAxislength -0.206082348
1og.sigma.1.0.mm.3D firstorder 9oPercentile -0.704269939
1og.sigma.3.0.mm.3D glrlm RunVariance -0.265190656
log.sigma.3.0.mm.3D glszm largeAreaHighGraylevelEmphasis 0.265449522
wavelet.llH firstorder Kurtosis -0.001610851
wavelet.lHl firstorder Mean -0.129011786
wavelet.HHl glszm SizezoneNonUniformityNormalized 0.110737160
wavelet.HHH firstorder Median -0.141196645

Calculation formula

$$RAD.scoreraw = \sum(featurei \times coefi)$$

$feature_i$:the value of the image feature; $coef_i$: COX regression coefficients

$$\mathbf{RAD.scorenorm} = \frac{\mathbf{RAD.scoreraw - RAD.scoremin}}{\mathbf{RAD.scoremax - Rad.scoremin}} \times \mathbf{100}$$

$RAD.score_{max}$ and $RAD.score_{min}$ are $RAD.score_{raw}$'s maximum and minimum values

Figure S1 Study flow diagram

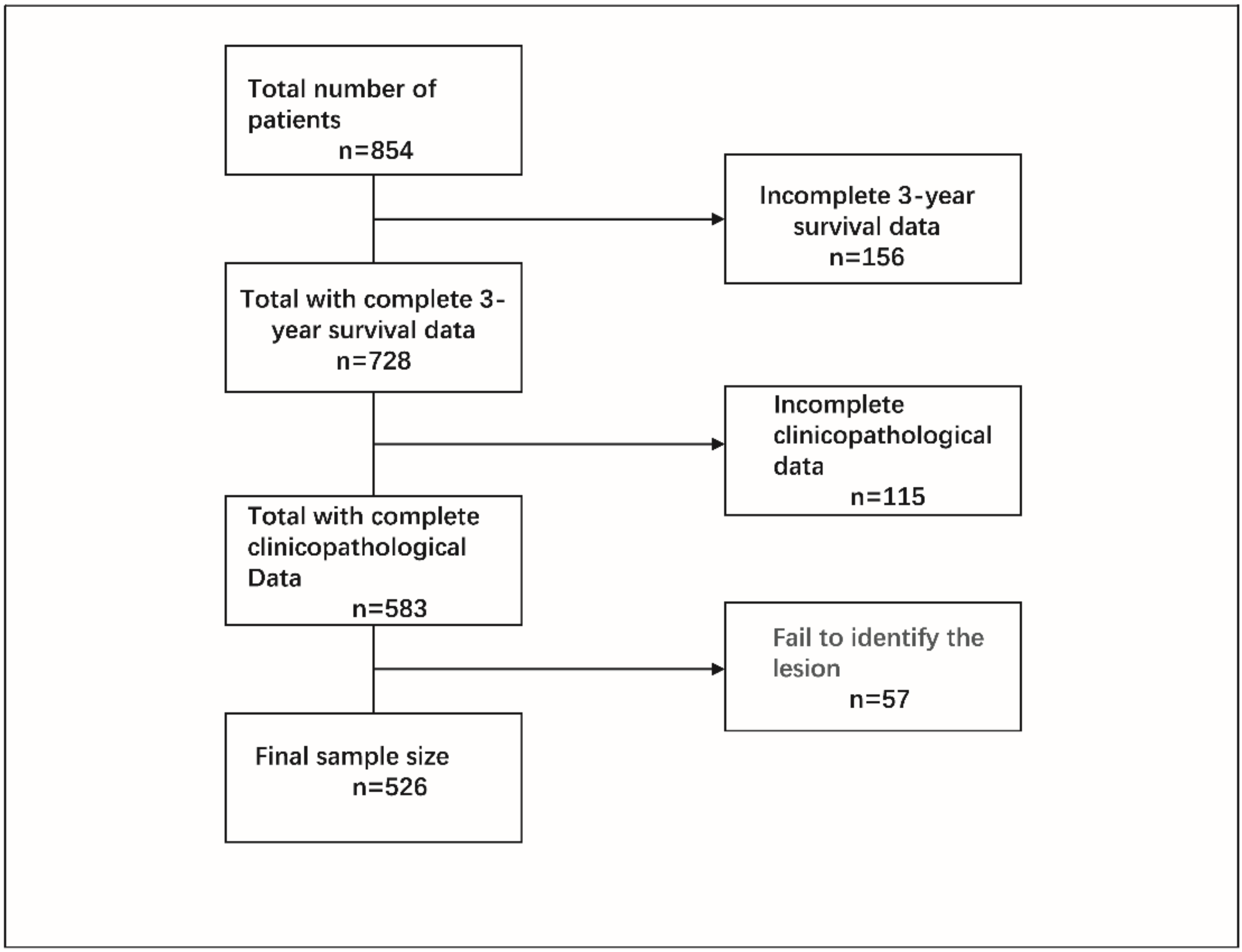

Figure S2 Selection of radiomics features via the LASSO regression algorithm

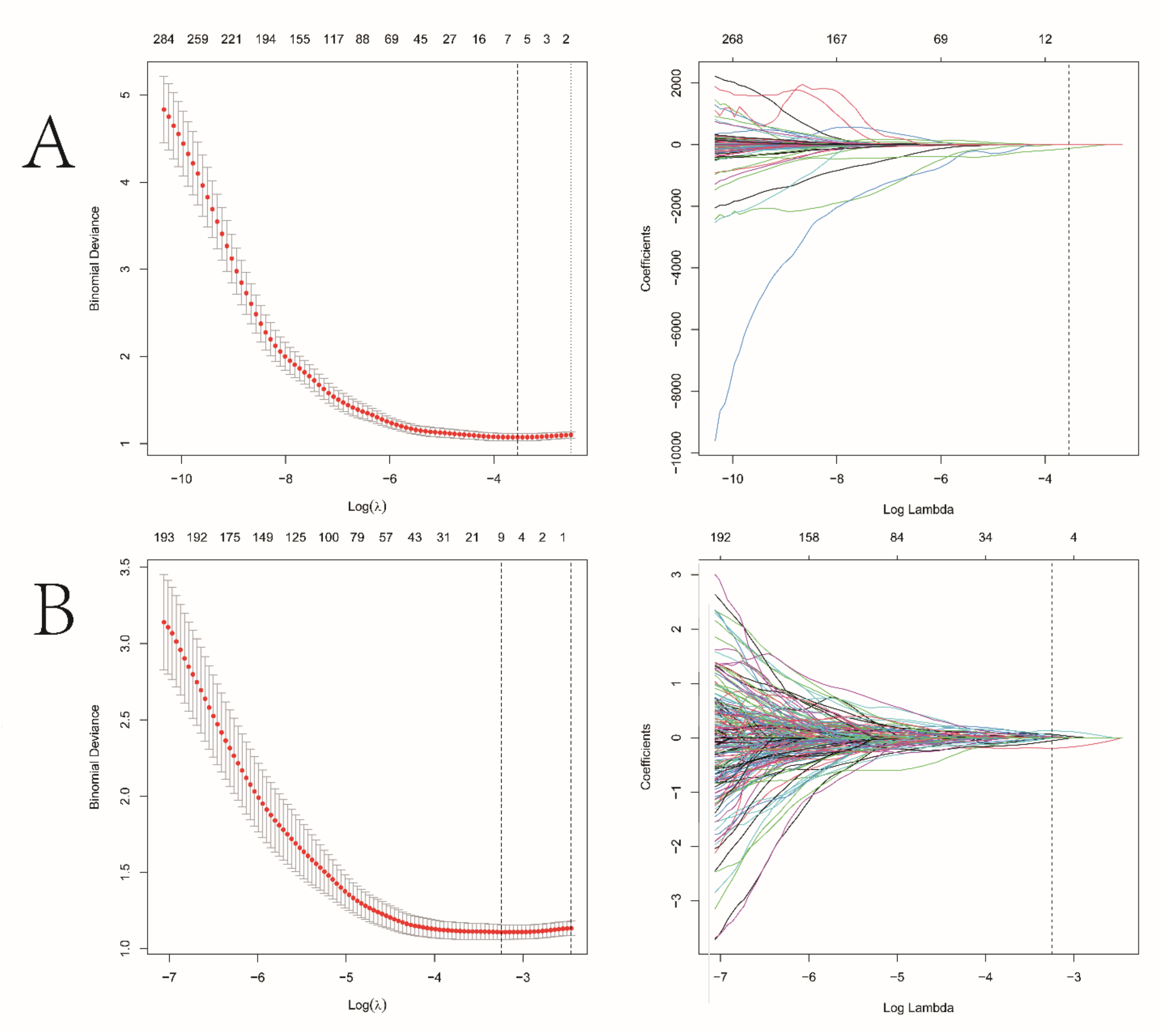

A: In the machine learning model, the optimal value of the LASSO tuning parameter (λ) is represented by a vertical dashed line (right line), and the value λ is selected as 0.029; LASSO coefficient curves for 302 radiomics features. A coefficient profile with the selected logarithmic λ value was generated using 10x cross-validation. Finally, six radiomics features with non-zero coefficients were selected.

B: In the nomogram, a value λ of 0.039 was chosen; LASSO coefficient profiles of the 302 radiomics features. A coefficient profile plot was generated versus the selected log λ value using tenfold cross--validation. Nine radiomics features with non--zero coefficients were finally selected.

Table S1 Screen variables that are meaningful to predictions through COX models

	B	SE	Wald	df	Sig.	Exp(B)	95.0% CI for Exp(B) Lower	Upper
Sex	-0.044	0.248	0.032	1	0.858	0.957	0.588	1.556
Age	0.007	0.013	0.258	1	0.612	1.007	0.981	1.033
LNPR	3.505	0.794	19.516	1	0.000	33.298	7.030	157.705
Differentiation status			10.777	2	0.005			
Moderate to well	0.923	0.601	2.358	1	0.125	2.516	0.775	8.171
Poor to moderate	1.444	0.605	5.687	1	0.017	4.237	1.293	13.881
LVI	-0.016	0.217	0.005	1	0.942	0.984	0.643	1.507
Tumor legth	0.183	0.058	10.102	1	0.001	1.201	1.073	1.344
CEA	-0.036	0.041	0.756	1	0.384	0.965	0.890	1.046
EO%	-0.060	0.049	1.503	1	0.220	0.942	0.856	1.037

LNPR: Lymph node positivity rate; LVI: Lymphatic vascular infiltration;

CEA: Carcinoembryonic antigen; EO%： Eosinophil percentage

Figure S3 Accuracy of Radiomics model and Clinicopathological data model for predicting recurrence

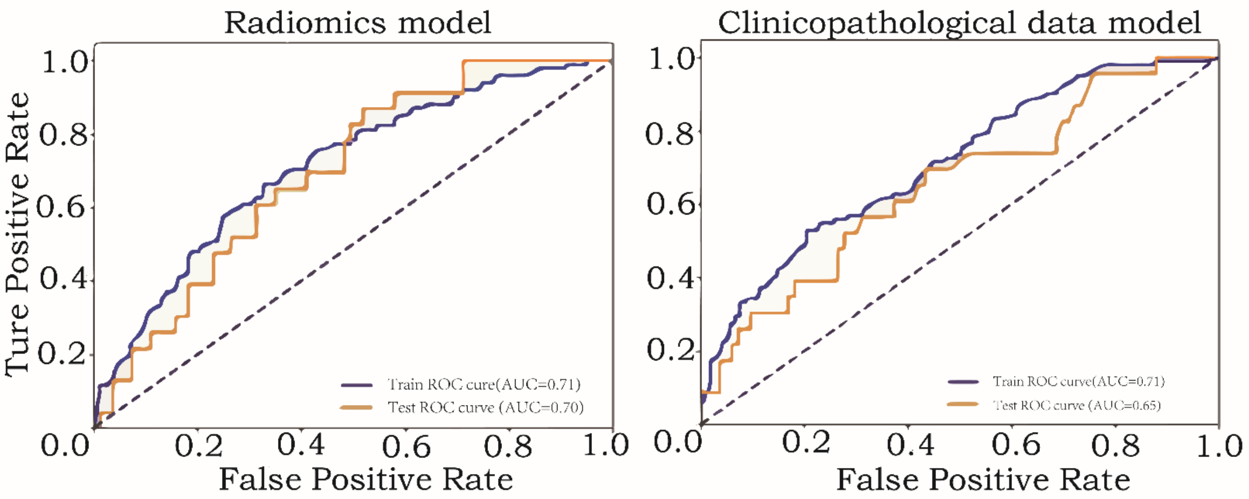

Figure S4-S6 Disease-free survival and overall survival between the recurrent and non-recurrent groups in all subgroups

“0”= no recurrent group “1”= recurrent group

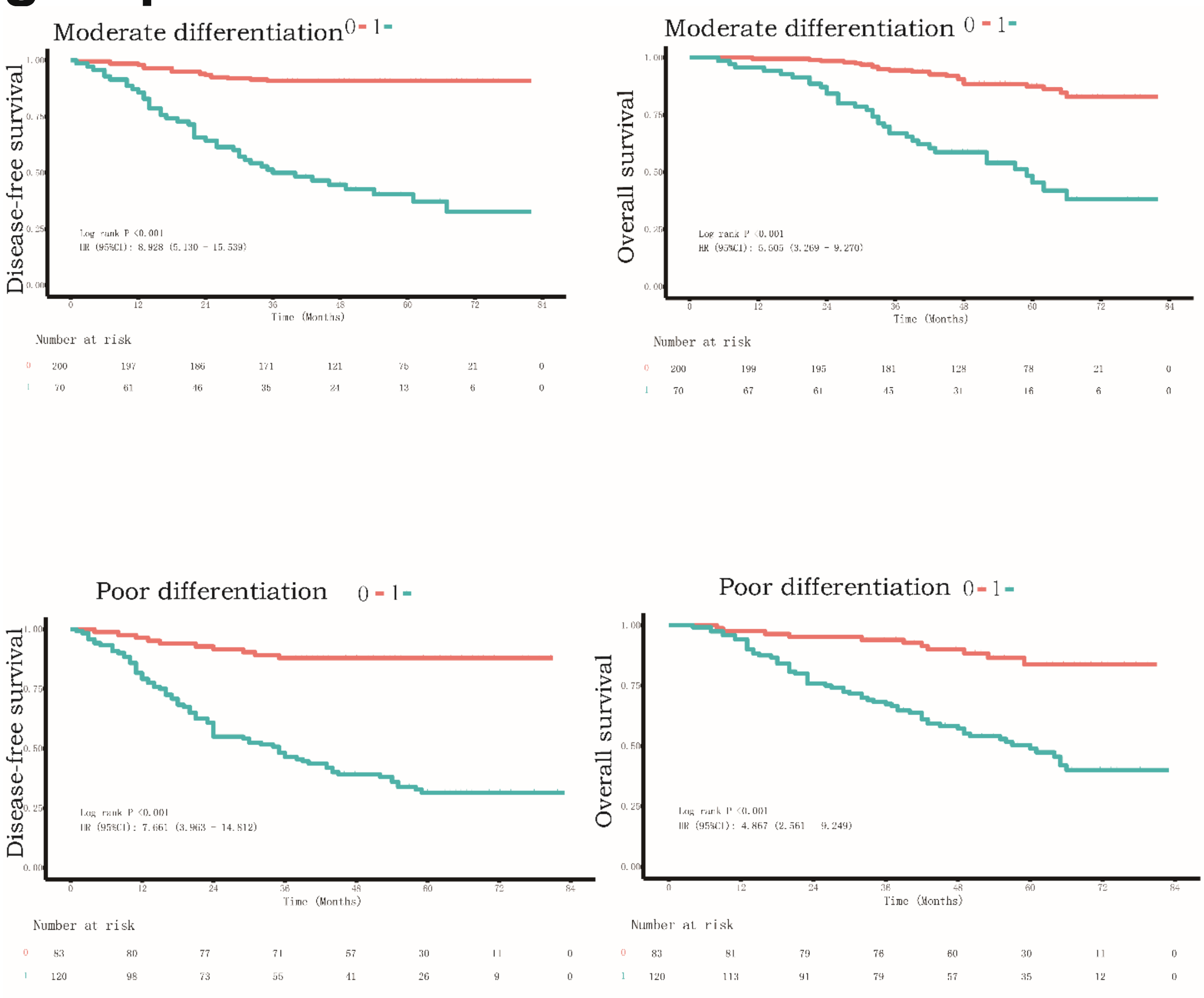

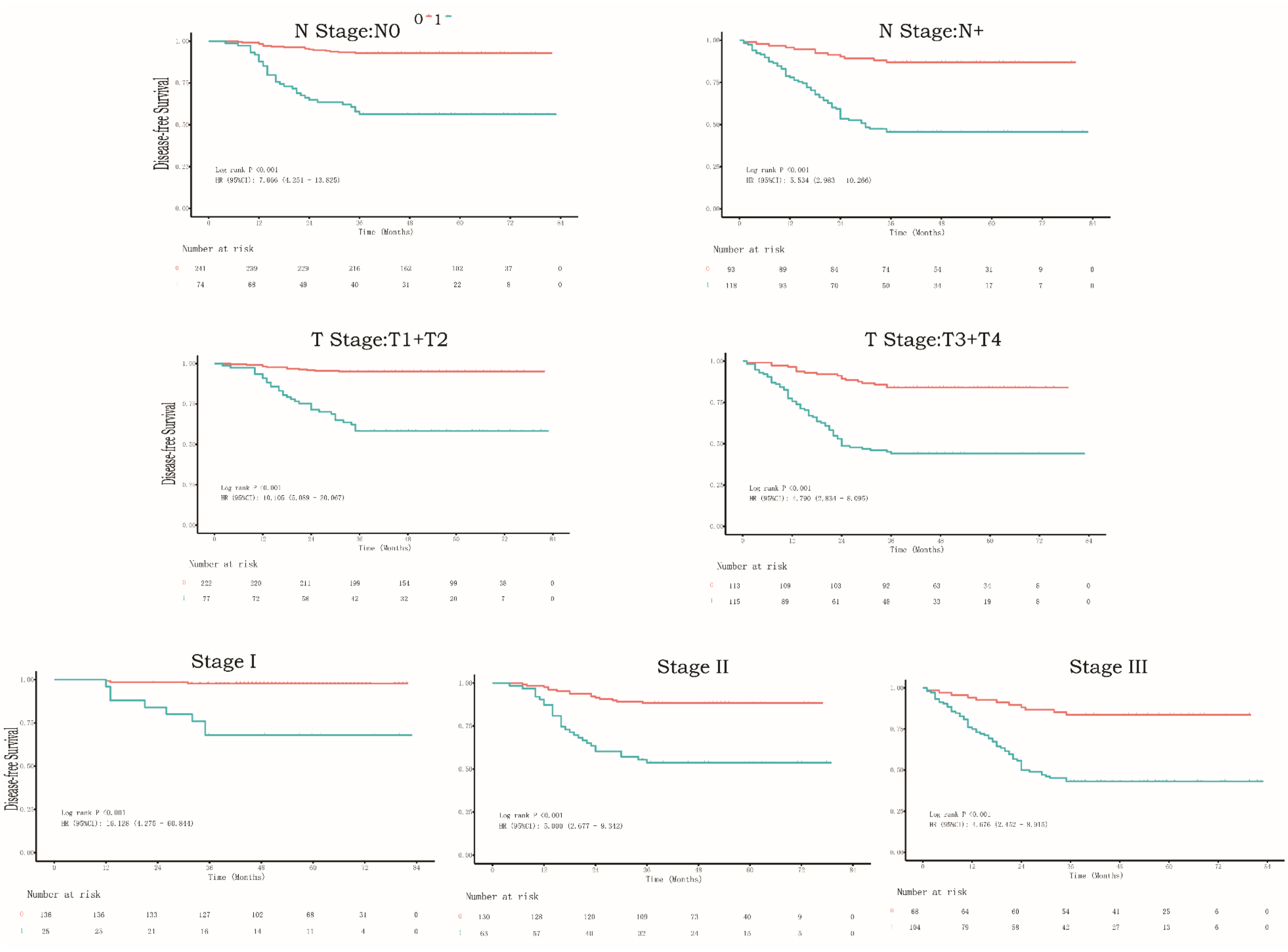

Insights Imaging (2025) Liu MY, Lu RX, Wang B, et al.

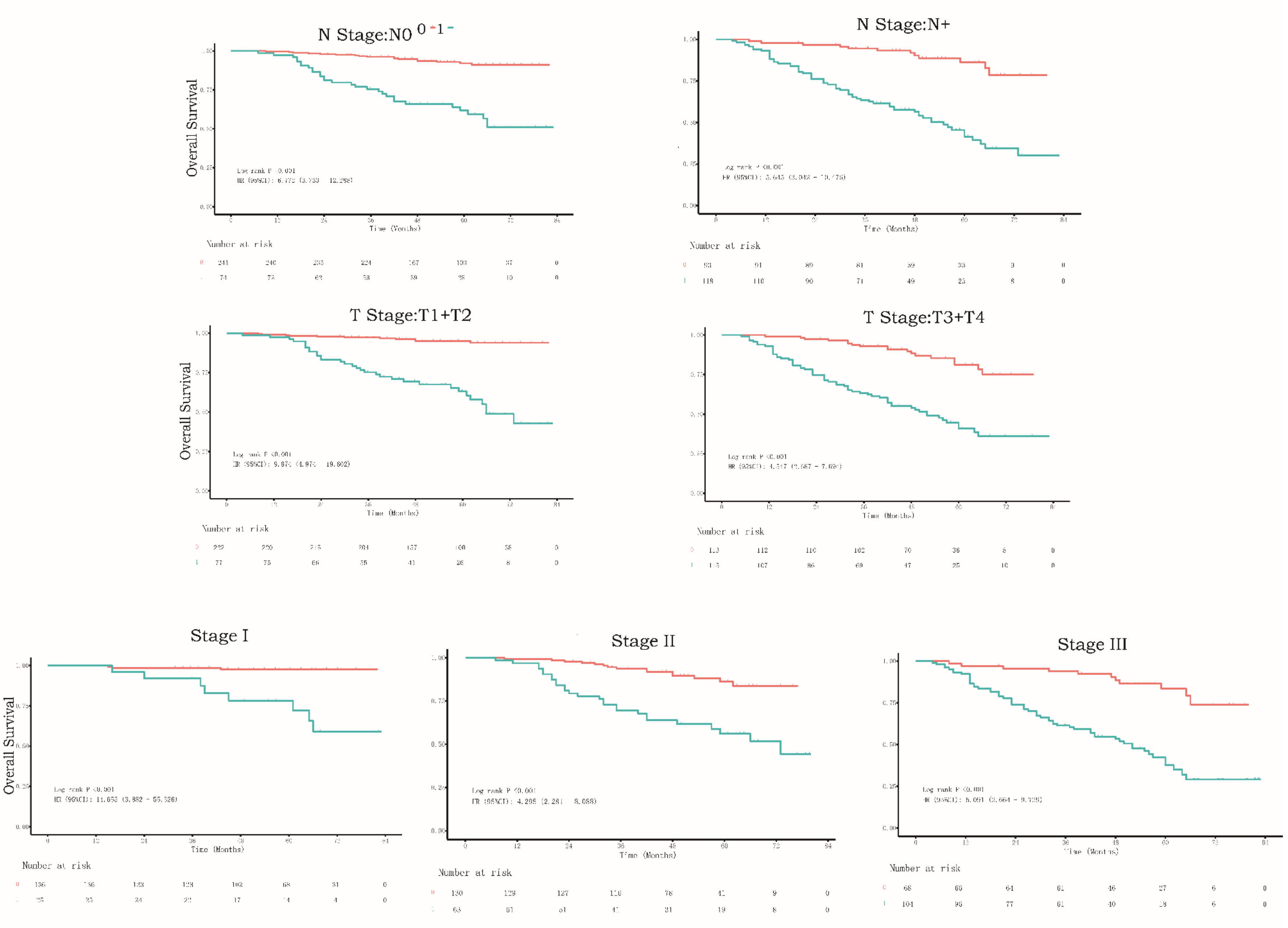

Insights Imaging (2025) Liu MY, Lu RX, Wang B, et al.

Figure S7 Decision curve analysis for the nomogram

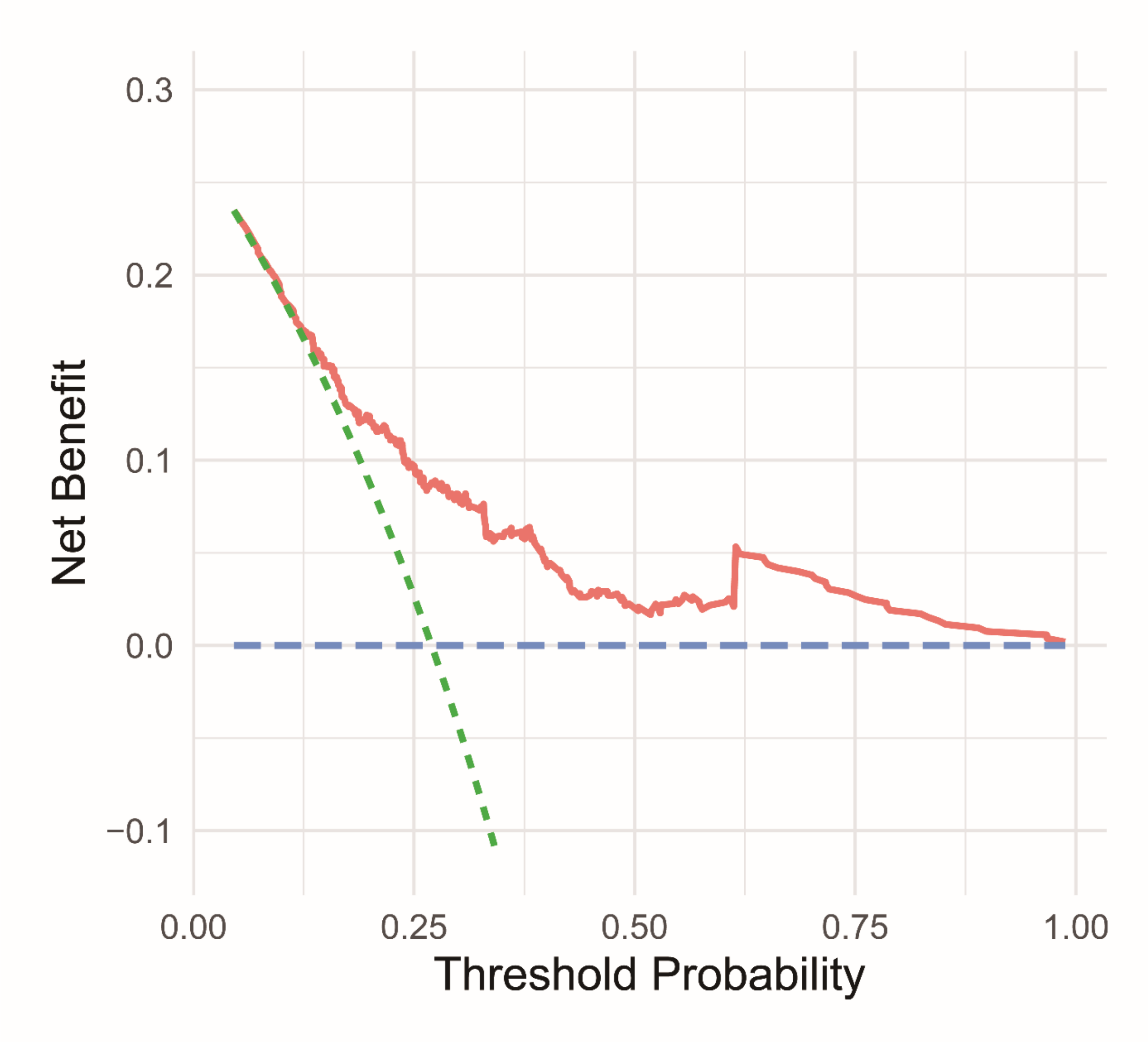

The abscissa is the threshold probability; the ordinate is the net benefit of the pros minus the cons; the blue horizontal line indicates all samples were negative, all were untreated, and the benefit was zero; the Green backslash indicates all samples were positive and all were treated .The red line represents net benefit of the nomogram, it suggests that the nomogram is beneficial and relatively safe

Figure S8 Disease-free survival of patients in different risk stratifications after PSM
“0”=No postoperative adjuvant therapy “1”= Postoperative adjuvant therapy

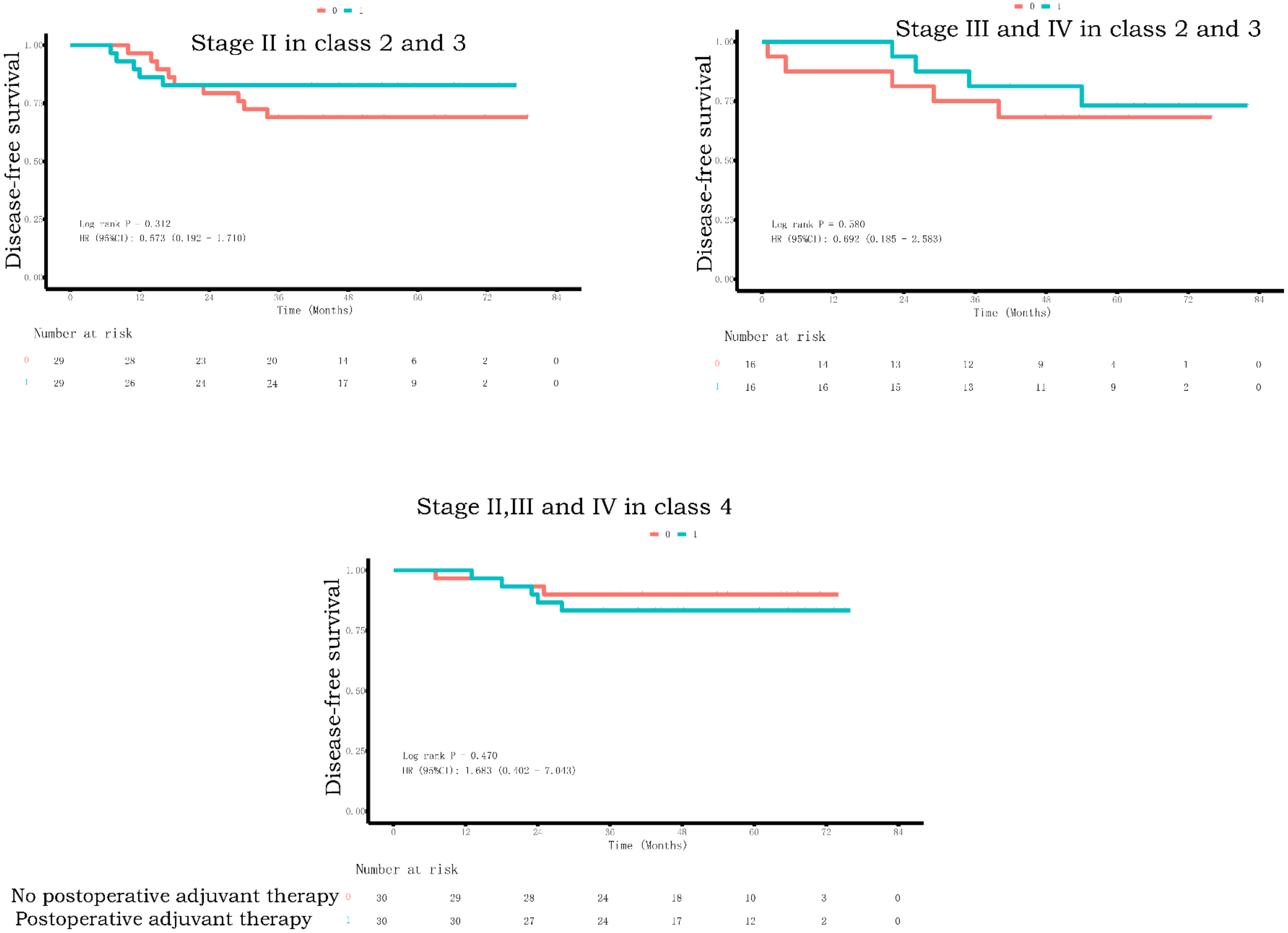

Supplement: Supplementary file 1 — ELECTRONIC SUPPLEMENTARY MATERIAL [file 13244_2025_2049_MOESM1_ESM.pdf]
